# Clonal reproduction of *Moniliophthora roreri* and the emergence of unique lineages with distinct genomes during range expansion

**DOI:** 10.1093/g3journal/jkad125

**Published:** 2023-06-20

**Authors:** Andrea Minio, Rosa Figueroa-Balderas, Stephen P Cohen, Shahin S Ali, Denny Carriel, Dahyana Britto, Conrad Stack, Indrani K Baruah, Jean-Philippe Marelli, Dario Cantu, Bryan A Bailey

**Affiliations:** Department of Viticulture and Enology, University of California Davis, Davis 95616, CA, USA; Genome Center, University of California Davis, 95616 Davis, CA, USA; Department of Viticulture and Enology, University of California Davis, Davis 95616, CA, USA; Sustainable Perennial Crops Laboratory, USDA/ARS, Beltsville 20705, MD, USA; Sustainable Perennial Crops Laboratory, USDA/ARS, Beltsville 20705, MD, USA; Mars La Chola (MLCH), Mars Inc., Guayaquil 090103, Ecuador; Mars Center for Cocoa Science, Mars Inc., Fazenda Almirante, Caixa Postal 55, Itajuípe, BA, CEP 45630-000, Brazil; Mars Digital Technologies, Mars Inc., Chicago 60642, IL, USA; Sustainable Perennial Crops Laboratory, USDA/ARS, Beltsville 20705, MD, USA; Mars Wrigley Plant Sciences Laboratory, Mars Inc., 95616 Davis, CA, USA; Department of Viticulture and Enology, University of California Davis, Davis 95616, CA, USA; Sustainable Perennial Crops Laboratory, USDA/ARS, Beltsville 20705, MD, USA

**Keywords:** cacao frosty pod, genome evolution, mating type loci, pathogenomics, *Theobroma cacao*

## Abstract

The basidiomycete *Moniliophthora roreri* causes frosty pod rot of cacao (*Theobroma cacao*) in the western hemisphere. *Moniliophthora roreri* is considered asexual and haploid throughout its hemibiotrophic life cycle. To understand the processes driving genome modification, using long-read sequencing technology, we sequenced and assembled 5 high-quality *M. roreri* genomes out of a collection of 99 isolates collected throughout the pathogen's range. We obtained chromosome-scale assemblies composed of 11 scaffolds. We used short-read technology to sequence the genomes of 22 similarly chosen isolates. Alignments among the 5 reference assemblies revealed inversions, translocations, and duplications between and within scaffolds. Isolates at the front of the pathogens' expanding range tend to share lineage-specific structural variants, as confirmed by short-read sequencing. We identified, for the first time, 3 new mating type A locus alleles (5 in total) and 1 new potential mating type B locus allele (3 in total). Currently, only 2 mating type combinations, A1B1 and A2B2, are known to exist outside of Colombia. A systematic survey of the *M. roreri* transcriptome across 2 isolates identified an expanded candidate effector pool and provided evidence that effector candidate genes unique to the *Moniliophthoras* are preferentially expressed during the biotrophic phase of disease. Notably, *M. roreri* isolates in Costa Rica carry a chromosome segment duplication that has doubled the associated gene complement and includes secreted proteins and candidate effectors. Clonal reproduction of the haploid *M. roreri* genome has allowed lineages with unique genome structures and compositions to dominate as it expands its range, displaying a significant founder effect.

## Introduction

The basidiomycete *Moniliophthora roreri* (Mr; causal agent of frosty pod rot) is a major pathogen of cacao (*Theobroma cacao*) originating in South America. *Moniliophthora roreri* is a hemibiotroph with an extended biotrophic phase of 45 days or longer (Evans 2016a; Bailey, Evans *et al.* 2018) and is considered an asexual haploid organism (Evans 2016a). Pod malformation may be the only external symptom of infection during the biotrophic phase. After the biotrophic phase, rapid necrosis occurs, leading to the formation of masses of rhexolytic thallic conidia on the surface of the infected pods (Díaz-Valderrama and Aime 2016b). A single infected cacao pod is capable of producing as many as 7 billion spores (Campuzano Londoño 1976). Spores are spread by wind and rain and, if unrestricted, infect and severely reduce yields. The life cycle of *M. roreri* is contrasted with that of its sister species, *Moniliophthora perniciosa*. *Moniliophthora perniciosa* biotype C is a sexual (homothallic) hemibiotroph causing the disease witches' broom of cacao, also originating in South America. *Moniliophthora perniciosa* produces basidiocarps, the resulting spores of which infect diverse meristematic tissues including pods, flower cushions, and branches (Evans 2016b).

Genetic recombination among *M. roreri* isolates is limited due to the apparent lack of a sexual cycle (Ali *et al.* 2015; Díaz-Valderrama and Aime 2016a; Bailey, Ali *et al.* 2018). As a result, genetic change in *M. perniciosa* is expected to be driven by mutations and chromosome rearrangements that are clonally replicated in subsequent generations. Due to its clonal reproduction, *M. roreri* displays limited diversity at the front of its expanding range in the western hemisphere and is strongly influenced by founder effects as it continues to spread. For example, the nations in Central America have been colonized by genetically similar *M. roreri* isolates following primarily a single line of descent (Phillips-Mora 2003). These isolates share similar SNP (Ali *et al.* 2015) and SSR (Díaz-Valderrama and Aime 2016a) profiles in addition to carrying A1B1 mating type alleles (Díaz-Valderrama et al. 2022). Though asexual, *M. roreri* carries the A and B loci required for tetrapolar mating, but only 2 allelic combinations (A1B1 and A2B2) were identified (Díaz-Valderrama *et al*. 2022).

We have a reasonable understanding of *M. roreri* genetic diversity, distribution, and the timing of its spread into new geographic regions (Evans 2016a); however, further investigation is needed to deepen our understanding of the species evolution as it expands to new regions in the absence of sexual reproduction. Our objectives include the assembly of chromosome-scale genomes for multiple genetically and geographically diverse isolates of *M. roreri*, the structural comparisons of these genomes within the species, and the characterization of processes driving genome modifications and adaptations associated with geographic dispersion. Better genome assemblies will be a fundamental resource of information to expand our understanding of *M. roreri*, in particular the impact of clonal propagation on genome evolution. Its clonal or near clonal reproduction habits, when combined with recent geographic expansion, allow for potential independent genome evolution due to unique genome structural modifications and mutations. This analysis will also help us gauge *M. roreri*'s potential for change and adaptation to parallel changes in their cacao host as a result of the selection and release of new breeding materials carrying tolerance to infection by *M. roreri*.

## Materials and methods

### *M. roreri* (Mr) and *M. perniciosa* (Mp) isolates

Most of the *M. roreri* isolates used in this study were from a collection held by the United States Department of Agriculture (USDA), Beltsville, USA. These isolates were collected from *T. cacao* fruit in affected areas of South and Central America between 1999 and 2013 (Supplementary Table 1). A SNP study (Ali *et al.* 2015) separated the diversity found in these isolates into 37 distinct genotypes including 14 “synonymous groups” with multiple members showing identical SNP profiles. Three isolates were collected more recently (2019–2020) from Ecuador (Supplementary Table 1). Isolates were selected for sequencing based on the original geographic location (i.e. represent all regions) and the genetic distance among isolates (i.e. maximize genetic diversity) (Ali *et al*. 2015). This resulted in isolates from 5 countries (2 from Costa Rica, 14 from Colombia, 4 from Ecuador, 1 from Peru, and 1 from Bolivia) and 8 of 14 SNP synonymous groups being included (Ali *et al*. 2015). The *M. roreri* cultures were isolated and maintained as previously described (Ali *et al.* 2015). *Moniliophthora perniciosa* isolate MCCS1 was collected in Bahia, Brazil, and maintained there in a similar manner.

### Isolation of genomic DNA for short-read and single-molecule real-time (SMRT) sequencing

For DNA extraction, *M. roreri* and *M. perniciosa* isolates were grown for 7 days on 20% clarified V8 agar (CV8). From the different cultures, 2–3 agar plugs (0.25 cm^2^) were transferred to 50-mL falcon tubes containing 20 mL liquid CV8 and grown at 25°C while shaking at 100 rpm. Cultures were incubated for 5 days and homogenized using a Janke & Kunkel Ultra Turrax T25 (IKA, Staufen, Germany) for 30–60 s. The homogenized mycelia (5 ml) were used as starter culture and added to 50 mL liquid CV8 in a 250-mL flask. Mycelia of each isolate were produced by growth at 25°C while shaking at 100 rpm for 7–10 days. The mycelia were washed with sterile water and collected by centrifuging at 20,000 g for 10 min, followed by flash freezing in liquid nitrogen and freeze-dried. Genomic DNA (gDNA) for the short-read and high molecular weight gDNA for SMRT sequencing were extracted from freeze-dried mycelia as previously described in Morales-Cruz *et al*. (2020). The gDNA concentrations were quantified with a Qubit 3.0 fluorometer using a Qubit dsDNA HS Assay Kit (Thermo Fisher Scientific, USA). The quality of the extracted gDNA was assessed using a NanoDrop spectrophotometer and 1% (*w*/*v*) agarose gel. The high molecular weight gDNA was run on a 0.75% pippin pulse (Sage Science, USA) gel to examine the integrity and molecular weight.

### Whole genome library preparations

A total of 22 *M. roreri* isolates from South and Central America (Supplementary Table 1) were subjected to whole genome Illumina sequencing. A total of 1 μg DNA per sample was used as the input material. DNA-seq libraries were prepared using the Kapa LTP library prep kit (Kapa Biosystems, MA, USA). Libraries were evaluated for quantity and quality with the High Sensitivity chip on a Bioanalyzer 2100 (Agilent Technologies, CA, USA) and sequenced in paired-end 150 bp reads on an Illumina HiSeq4000 (Novogene Corporation Inc, Sacramento, CA, USA).

The genome of *M. perniciosa* MCCS1 and 5 of the *M. roreri* isolates were subjected to SMRT sequencing (Table 1 and Supplementary Table 2). High molecular weight gDNA was cleaned with 0.45 × AMPure PB beads (Pacific Biosciences, Menlo Park, CA, USA) before library preparation. SMRTbell template was prepared with 15 µg of sheared DNA using SMRTbell Template Prep Kit (Pacific Biosciences, Menlo Park, CA, USA) following the manufacturer's instructions. SMRTbell template was size-selected using the BluePippin instrument (Sage Science, Beverly, MA, USA) using a cutoff size of 17–50 Kbp. The size-selected library was cleaned with 1 × AMPure PB beads followed by a DNA damage repair treatment and cleaned again with 1 × AMPure PB beads. One SMRTbell library was produced for each of the 5 genotypes, which were sequenced on separate SMRT cells on the PacBio Sequel II platform (DNA Technology Core Facility, University of California, Davis).

**Table 1.** Genome sequencing, assembly, and annotation statistics for the 5 *M. roreri* isolates (MrB3, MrCo8, MrCo84, MrE7, and MrC26) and the *M. perniciosa* isolate (Mp-MCCS1) sequenced using long-read technology sequencing.

Metrics	MrB3	MrCo8	MrCo84	MrE7	MrC26	Mp-MCCS1
Sequencing statistics						
Sequencing data (Gbp)	129	15	140	79	9	128
Coverage (X-fold)	2,144×	242×	2,341×	1,317×	156×	2,600×
Assembly statistics						
Cumulative length (Mbp)	57.48	58.18	56.81	57.39	59.57	48.97
Number of contigs	11	11	13	21	11	12
Average sequence length (Mbp)	5.22	5.29	4.37	2.73	5.42	4.08
Maximum sequence length (Mbp)	10.74	10.4	10.41	10.25	10.45	9.61
N50 length (Mbp)	5.18	5.19	4.88	4.88	5.17	5.46
N90 length (Mbp)	3.46	3.58	3.61	3.49	3.58	3.55
Annotation statistics						
Number of genes	20,704	21,007	20,409	20,539	21,460	17,726
Repetitive content	17.84%	17.59%	17.51%	17.90%	18.89%	10.45%

Isolate MrC26 was also sequenced with additional long-read sequencing using Oxford Nanopore technology. High molecular weight gDNA was cleaned, and libraries were produced (Oxford Nanopore, Oxford, UK) and sequenced using Nanopore MinION technologies. The run produced a total of 2.05 Gbp (∼35× X-fold coverage) in 787,832 reads with an N50 of 6.1 Kbp.

### Genome assembly

Genome size was estimated by k-mer content analysis of short-read libraries. K-mer count and frequency were calculated with Jellyfish (ver. 2.2.7, Marçais and Kingsford 2011) using a k-mer length of 21 bp. Genome haploid length and heterozygosity rates were estimated with GenomeScope (ver. 2.0, Ranallo-Benavidez *et al.* 2020) by fitting the k-mer distribution to a mixture model of 4 evenly spaced negative binomial distributions to the k-mer profile.

PacBio reads sequenced for MrC26, MrB3, MrCO84, MrCO8, MrE7, and M. *perniciosa* MCCS1 genotypes were assembled separately using Falcon (ver. 2017.06.28–18.01, Chin *et al.* 2016) assembler testing multiple parameter combinations to achieve the lowest fragmentation. The procedure took advantage of repetitive content identification in both raw and error corrected reads as described in Minio *et al.* (2019). Assembled contigs were then polished using Arrow (from ver. GCpp 1.0.0-cd36561, “https://github.com/PacificBiosciences/gcpp”). MrC26 and Mp-MCCS1 underwent a scaffolding procedure with SSPACE-LongRead (ver. 1.0, Boetzer and Pirovano 2014). MrC26 scaffold structure was then validated with independent 10 × using Tigmint (ver. 1.2.4, Jackman *et al.* 2018) and NanoPore sequencing data assembled using CANU (ver. 1.8–14, Koren *et al.* 2017), Miniasm (ver. 0.3-r179, Li 2016), Shasta (ver. 0.3.0, Shafin *et al.* 2020), Flye (ver. 2.6-g0d65569, Lin *et al.* 2016; Kolmogorov *et al.* 2019), RA (ver. 0.2.1, “https://github.com/lbcb-sci/ra”), and wtdbg2 (ver. 2.3, Ruan and Li 2019). The structure of the scaffolds was then compared with the contigs of the other genotypes to identify syntenic groups of sequences. Pairwise comparisons of all assemblies were performed by aligning the scaffolds of contigs sequences with nucmer (MUMMER v4, ver. 4.0.0beta5, Marçais *et al.* 2018), local hits were then inserted in an adjacency network, and the highest coverage tiling paths of nonoverlapping hits were identified between all pairs of sequences. Each sequence of each draft assembly was then assigned to a single homolog sequence in each other assembly in a 1-to-1 relationship selecting the overall best of the tiling paths. Pairwise relationships were used to identify syntenic groups and, when possible, draft sequences belonging to the same pseudomolecule as assembled in a single contig in a different genotype.

Illumina reads of each genotype underwent clipping of adapters and quality trimming with Trimmomatic (ver. 0.36, parameters: “ILLUMINACLIP:2:30:10 LEADING:7 TRAILING:7 SLIDINGWINDOW:10:20 MINLEN:36,” Bolger *et al.* 2014) assembled independently using SPAdes (ver. 3.13.0 edited to allow k-mers up to 251, Bankevich *et al.* 2012). Different k-mer combinations were tested, and a multiple k-mer set of 105, 115, 125, 135, and 145 was then used to produce the assemblies for each genotype. Assembly completeness was evaluated using BUSCO (v.5, Agaricales odb10 database, Supplementary Table 3, Simão *et al.* 2015).

Error rates and heterozygosity estimation were performed by calling variants between each genome assembled with SMRT reads and Illumina reads produced for the very same genotype. Short reads were aligned using BWA MEM (ver. 0.7.17, Li 2013) and randomly downsampled to 200 × of coverage with Samtools (ver. 1.9, Li *et al.* 2009). Alignments were then postprocessed with Picard tools (ver. 2.12.1-SNAPSHOT, Broad institute) to assign reads groups and mark duplicates and variants called. Variant calling was then performed using GATK HaplotypeCaller (ver. 4.0.12.0, parameters: “–ERC GVCF –base-quality-score-threshold 20 –sample-ploidy 2,” Poplin *et al*. 2017) and genotyped with GenotypeGVCFs (parameters: “–include-non-variant-sites true –sample-ploidy 2”).

To establish phylogenetic relationships among the *M. roreri* strains, variant sites were collected from nonrepetitive regions of the genome where all samples were genotyped with a depth of coverage 5 ≤ dp ≤ 1000. Phylogeny was calculated with RAxML-NG (ver. 0.9.0, parameters: “–model GTR + G + FO –tree pars{5} –bs-trees 100 –seed 12345,” Kozlov *et al*. 2019), and tree graph was then produced with FigTree (ver. 1.4.4, “http://tree.bio.ed.ac.uk/software/figtree/”).

### Generation of new and acquisition of existing RNA-Seq data

The *M. roreri* RNA-Seq data used were derived from 3 sources: mycelia grown in vitro (this study), artificially inoculated cacao pods (Meinhardt *et al.* 2014), and naturally infected cacao pods (Bailey *et al*. 2014).

For RNA extraction from mycelia, *M. roreri* isolate C26 was grown as mentioned above. RNA from freeze-dried mycelia were extracted following a described earlier procedure (Bailey *et al.* 2013). RNA purity was determined using a NanoDrop 2000 spectrophotometer (Thermo Scientific, Hanover Park, IL). The RNA quantity was determined using the RNA broad range kit of the Qubit 2.0 Fluorometer (Life Technologies, Carlsbad, CA). RNA integrity was determined by electrophoresis using an Agilent 2100 Bioanalyzer (Agilent Technologies, CA). Total RNA (300 ng, RNA integrity number > 8.0) was used for cDNA synthesis and library construction. Three RNA-Seq libraries were prepared using the Illumina TruSeq RNA sample preparation kit v.2 (Illumina, CA, USA) following Illumina's low-throughput protocol. This library was evaluated for quantity and quality with the High Sensitivity chip in an Agilent 2100 Bioanalyzer (Agilent Technologies, CA) and was sequenced in 150 bp paired-end reads, using an Illumina HiSeq4000 sequencer (Novogene Corporation Inc, Sacramento, CA, USA).

The RNA-Seq data for artificially inoculated cacao pods at 30 days post infection (DPI) and 60 DPI were obtained from a previously published study (Meinhardt *et al.* 2014). Data for 3 independent biological replicates for each of the time points were included in the analysis (NCBI BioProject PRJNA229176). Information related to the *M. roreri* infection assay, RNA isolation, and RNA-Seq analysis can be obtained from the previous study (Meinhardt *et al.* 2014). Based on the typical progression of *M. roreri* infection, malformed green pods were selected for RNA-Seq at 30 DPI, corresponding to the biotrophic phase. Necrotic sporulating pods were selected for RNA-Seq at 60 DPI to represent the necrotrophic phase.

The third source of RNA-Seq data was previously discussed in Bailey *et al.* (2014). The dataset comprises 12 independent cacao pods from 4 different cacao clones (3 per clone) naturally infected and representing a range of symptoms.

*Moniliophthora perniciosa* mycelia were produced using 15-mm disc plugs from a 5-day-old culture grown on potato dextrose agar (PDA) [20% potato (*w*/*v*), 0.2% dextrose (*w*/*v*), and 1.5% agar (*w*/*v*)] which were transferred to 250 mL potato dextrose broth and incubated in the dark for 4 days at 25°C, shaking at 110–125 rpm orbital shaking. Subsequently, 20 mL mycelia culture was homogenized by Polytron probe for 60 and 10 mL transferred to 250 mL potato dextrose broth and incubated as before. Finally, the entire fungus culture was filtered, and the biomass was washed with sterile distilled water, frozen with liquid nitrogen, and lyophilized.

Biomass of basidiocarps was produced as described by Griffith and Hedger (1993) with some modifications proposed by Macagnan *et al.* (2006). Briefly, preincubation of the fungus was made at 25°C for 15 days on PDA. Afterwards, agar plugs were transferred to autoclaved compacted bran-based solid medium containing 68 g of triturated cacao dry broom, 18.2 g of oat flakes, and 2.75 g of CaSO_4_ and brought to 100 mL with distilled water. Once produced, basidiocarps were collected and washed with sterile distilled water, frozen with liquid nitrogen, and lyophilized. Basidiospores were collected from the basidiocarps according to the method of Frias *et al.* (1995) with some modifications. Before fixing the pile on the Petri dish, the basidiocarps were gently disinfected with a 0.1% streptomycin solution, washed with sterile distilled water, and dried on a paper towel. Basidiospore collection was carried out overnight at 25°C, stirring at 110 rpm.

Biomass of green brooms and necrotic brooms was produced after inoculation as described by Sena et al. (2014). Briefly, apical shoot apexes of 4-week-old seedlings from cacao genotype were inoculated with a 30 μl drop of a basidiospore suspension (2 × 10^5^ basidiospores/mL with >80% germination) in 0.3% agar (Surujdeo-Maharaj *et al.* 2003). After inoculation, 30 days for green broom and 60 days for dry broom, infected branches were washed with distilled water, frozen in liquid nitrogen and lyophilized, and then preserved in vacuum plastic bags.

### Gene prediction

Custom repeat libraries were created for *M. roreri* and *M. perniciosa* genomes separately with RepeatMasker (ver. open-4.0.6, Smit et al. 2013) and RepeatModeler (ver. open-1.0.11, Smit and Hubley 2015) starting from homologs to known fungal transposable elements present in RepBase (ver. 20160829).

RNA-Seq reads underwent clipping of adapters and quality trimming with Trimmomatic (ver. 0.36, parameters: “ILLUMINACLIP:2:30:10 LEADING:7 TRAILING:7 SLIDINGWINDOW:10:20 MINLEN:36,” Bolger *et al.* 2014). Samples were assembled de novo separately using Trinity (ver. v2.2.0, Grabherr *et al.* 2013; Haas *et al.* 2013) and used as input to PASA (ver. v2.3.3, Haas *et al.* 2003, 2008, 2011) to produce a nonredundant training set of protein-coding gene models over MrC26 PacBio genome assembly for *M. roreri* isolates and Mp-MCCS1 for *M. perniciosa*. The resulting datasets were used in BRAKER (ver. 1.9, Hoff *et al.* 2016) to obtain ad hoc models for Augustus (ver. 3.0.3, Stanke *et al.* 2006). Ab initio prediction of protein-coding gene loci was produced for all PacBio and Illumina-based draft assemblies.

For *M. roreri* isolates, the collected coding sequences (CDS) of all identified models were then searched for transposable elements related proteins by BLASTp (ver. 2.7.1+, Altschul *et al.* 1990) search against RefSeq database (retrieved 2017_01_17) and clustered in a nonredundant set of transcripts using EvidentialGene (ver. 2014.05.15, Gilbert 2016). The nonredundant set of transcripts was then mapped with blat (ver. 36 × 2, Kent 2002) on the genomic sequences, and hit regions with identity > 80% and coverage > 50% to the original transcript not overlapping annotated loci were then analyzed with TransDecoder (ver. 3.0.1, “https://github.com/TransDecoder/TransDecoder”) to recover missing protein-coding gene annotations and identifying potential pseudogenes.

The proteins annotated on all genotypes were mapped against RefSeq database (retrieved 2017 January 17) using BLASTp (ver. 2.7.1+, Altschul *et al.* 1990) with coverage > 50% and identity > 50% to assign a functional description based on sequence homology. CAZyme annotation was performed using HMMer (ver. 3.1b2, Eddy 1998, hmmer.org) with dbCAN database (ver. 4, Yin *et al.* 2012). Secondary metabolites clusters in all genomes were searched using antiSMASH (ver. 6.0.0, Medema *et al.* 2011; Blin *et al.* 2019). For PacBio annotated genes, InterProScan was also performed to further annotate the proteins with functional domains and associate Gene Ontology terms in Blast2GO (ver. 5, Conesa *et al.* 2005).

### Structural variants (SVs), SNP, and short InDel identification among *M. roreri* isolates

To identify SVs across the different isolates, SPAdes de novo assemblies underwent pairwise comparison to MrC26, MrB3, MrCO84, MrCO8, and MrE7 Falcon assembly. Assembled sequences were mapped using nucmer (MUMMER v4, ver. 4.0.0beta5, Marçais *et al.* 2018) with default parameters, SNPs and short InDels were identified extracted with show-snps (parameters “-Clr -x 1 –T,” MUMMER v4, ver. 4.0.0beta5, Marçais *et al.* 2018), and SVs were identified with show-diff (MUMMER v4, ver. 4.0.0beta5, Marçais *et al.* 2018). For each reference genome, all variants identified across all query genotypes were collected to generate a catalog of breakpoints with a tolerance of 50 bp. Shared breakpoints across all genotypes were collected, and the binary distance between them was calculated as per Rogers and Tanimoto (1960) [S6 coefficient of Gower and Legendre, s4 = (a + d)/(a + 2(b + c) +d)] to generated dendrograms.

### Gene expression analysis

Filtered and quality trimmed RNA-Seq reads were mapped using Bowtie2 (ver. 2.3.4.1, parameters: “–non-deterministic -k 1 –very-sensitive –t,” Langmead and Salzberg 2012) on a reference transcriptome consisting of MrC26 and *Theobroma cacao* Matina1–6 genome v1.1 annotated transcripts. Read counts and differential expression analysis were performed in R using GenomicAlignments (v1.18.1, Lawrence *et al.* 2013) package and differential expression with DESeq2 package (Love *et al.* 2014). R implementation of Fisher's exact test (Fisher 1935) was used to calculate odd ratios and significance of upregulated genes in the subsets of all secretome genes, predicted effectors, secreted genes unique to *M. roreri*, secreted genes common and exclusive to *M. roreri* and *M. perniciosa*, or secreted genes in *Marasmiaceae*.

### Orthologous groups and protein-based phylogeny

Ortholog gene groups were identified with OrthoFinder (ver. 2.2.7, Emms and Kelly 2015) using as input BLASTp (ver. 2.7.1+, Altschul *et al.* 1990) protein-to-protein alignments among *M. roreri* genotypes only and with an outgroup composed of *M. perniciosa* isolate MCCS1, *Fusarium oxysporum forma specialis Lycopersici* 4286, *Marasmius fiardii* PR-910, *Rhizoctonia solani* AG-1 IA, and *Tetrapyrgos nigripes* CBS291.85. The species composing the outgroups were selected for increasing distance in the order of *M. perniciosa* (*Marasmiaceae*), *M. fiardii* PR-910 (*Marasmiaceae*), *T. nigripes* (*Marasmiaceae*), *R. solani* AG-1 IA (*Basidiomycota*), and *F. oxysporum forma specialis Lycopersici* 4286 (*Ascomycota*).

Single-copy ortholog genes were used to build the phylogenetic tree of the selected species. Proteins of each group underwent multiple alignment with MAFFT (ver 7.475, parameters: “–maxitrate 1000 –localpair,” Katoh and Standley 2013). The alignments were concatenated and trimmed with ClipKIT (ver. 1.4.1, Steenwyk *et al.* 2020). RAxML-NG (ver 0.9.0, cit., parameters: “–model LG + G8 + F –seed 12345 –tree pars{10} –bs-trees 200,” Kozlov *et al.* 2019) was used to build a phylogenetic tree. FigTree (ver. 1.4.4, “http://tree.bio.ed.ac.uk/software/figtree/”) was used to produce the graphical representation rooted on *M. perniciosa* MCCS1 sample as an outgroup.

### Secretome and effector prediction

Protein-coding sequences annotated in all *M. roreri* and *M. perniciosa* isolates were scanned for possible signal peptides using SignalP, version 5.0 (Almagro Armenteros *et al.* 2019). The amino acid sequences containing predicted signal peptides were scanned for transmembrane proteins using the TMHMM program (Sonnhammer *et al.* 1998). Proteins with no more than 1 transmembrane domain were considered potential components of the secretome. All candidate proteins were first clustered with CD-HIT (ver. 4.6.8-2017-0621, parameters: “-g 1 -c 0.99 -s 0.5 -aS 0.5 -p 1,” Li and Godzik 2006) to reduce the dataset redundancy, and cluster representatives were then self-mapped with BLASTp (ver. 2.7.1+, Altschul *et al.* 1990) and underwent Markov clustering with MCL (ver. 14–137, Enright *et al.* 2002) following methods described by Cantu *et al.* (2013).

Fungal effectors among the *M. roreri* secretome were predicted using the machine learning program EffectorP 2.0 and EffectorP 3.0 (Sperschneider *et al.* 2018; Sperschneider and Dodds 2022). Each secreted protein was searched for the effector motifs [L/I]xAR, [R/K]CxxCx12H, RxLR, [Y/F/W]xC, YxSL[R/K], and G[I/F/Y][A/L/S/T]R following methods described previously (Cantu *et al.* 2013). Secreted proteins with <150 amino acids long and a cysteine content higher than 3% were cataloged as small and cysteine rich. Each secreted protein was scored based on the EffectorP 2.0 and 3.0 prediction, similar to previously characterized effector proteins, presence of any effector motifs, absence of any annotation, and small cysteine-rich criteria. The scoring was done following methods previously described by Cantu *et al.* (2013). A combined score was then used to rank the tribes based on their likelihood of containing potential effector proteins.

### Mating type gene identification

Nucleotide CDS sequences were aligned to the genomes using GMAP (ver. 2019-09-12, Wu *et al.* 2016) and blat (ver. 36 × 2, Kent 2002), and protein sequences were compared with the annotated proteins by BLASTp (ver. 2.7.1+, Altschul *et al.* 1990) and to the genome with exonerate (ver. 2.2.0, Slater and Birney 2005). Concomitant matches were used to identify the annotated loci ascribed to the HD1, HD2, STE3_Mr4, and Mr_Ph4 mating type genes. CDS for Mr_Ph4 could not be found annotated in isolates MrCo58 and MrCo67. The genomic regions surrounding the B locus were manually inspected, and sequences encoding open reading frames corresponding to the Mr_Ph4 sequence position and length were used for MrCo58 and MrCo67.

The CDS of type genes were aligned with the CDS of mating type genes from Díaz-Valderrama and Aime (Díaz-Valderrama and Aime 2016a). Alignments were conducted with MUSCLE (ver. v3.8.31, Edgar 2004) using the nucleotide-coding sequences. Trees were constructed RAxML (ver. v.8.2.12, Stamatakis 2014) using the GTRGAMMA model with 1000 bootstrap replications.

## Results

### Assembly of long reads reveals *M. roreri* genome architecture

SMRT sequencing was used to produce genome assemblies for 5 *M. roreri* isolates from South and Central America (Table 1 and Supplementary Table 2). With an estimated sequencing depth ranging from 156× to 2,341×, the size of the assemblies produced was 57.89 ± 1.06 Mbp (Table 1). This closely matches the estimated genome size of 57.91 ± 1.95 Mbp based on k-mer distribution analysis. The assemblies contained an average of 20,824 ± 420 predicted protein-coding genes, and 18% of the sequence was identified as repetitive. The accuracy of the genome assemblies was confirmed through comparison with short-read sequences. On average, the genomic sequences had an error rate of 0.0197 ± 0.0076%. Heterozygous variants were rarely found in the genomes (<0.00069% of the bases), confirming isolates are haploid. The assemblies were 96.4 ± 0.3% complete according to BUSCO (Supplementary Table 3) and highly contiguous with an average N50 of 5.05 ± 0.17 Mbp. Sequences of each genome were grouped into 11 syntenic groups. A few contigs covering 0.2 and 1.1% of the MrCO84 and MrE7 genomes, respectively, could not be placed in any of the syntenic scaffolds (Table 1). For 6 of the syntenic groups, it was possible to identify telomeric repeats at both extremities of the sequence for at least 1 genotype, suggesting they may correspond to complete chromosomes. For the remaining 5 groups, telomeric repeats were found only at 1 end of the sequence. Twenty-two genetically diverse *M. roreri* isolates, collected over the pathogen's geographic range and including the 5 isolates sequenced by SMRT technologies (Supplementary Table 1), were also sequenced using short-read Illumina sequencing technologies (Supplementary Table 2). The average assembly size of Illumina reads was 58.03 ± 1.47 Mbp in 4,223 ± 1,058 contigs with an average contig length of 14,389 ± 2,774 bases (Supplementary Table 2). Analysis using BUSCO models indicated that 94.8 ± 0.1% of the genes are complete, indicating the overall comprehensiveness of these assemblies (Supplementary Table 3).

### Structural variability across *M. roreri* isolates

**Fig. 1.**
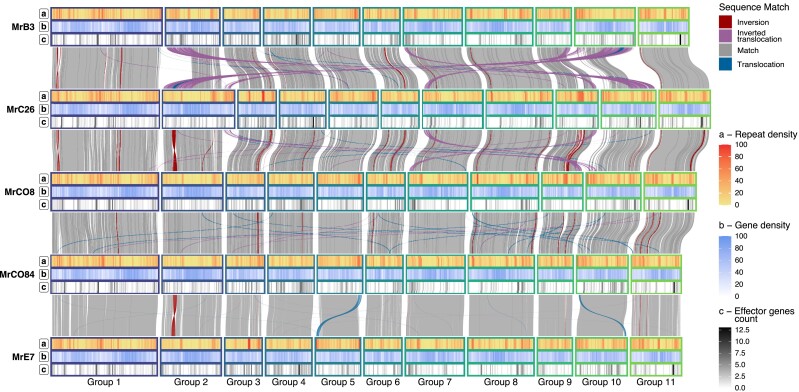
Colinearity between *M. roreri* isolates syntenic groups. Colinear regions between the 5 isolates have been depicted across the 5 different *M. roreri* isolates evidencing distant translocation events (blue) and inversions (red). For each of the genotypes, the 3 tracks reported indicate a) the local repeat density in terms of percentage of bases associated to a repetitive element, b) the local gene density in terms of bases ascribed to gene loci, and c) the effector gene density in terms of number of genes in a 100 Kb window.

Whole genome alignments of the 5 SMRT assemblies revealed structural rearrangements, primarily involving insertions and duplications (Fig. 1 and Supplementary Fig. 1). On average, variants were found to make up 5.78 ± 1.32 Mbp when comparing each pair of isolates, corresponding to 9.98 ± 2.29% of the total genome assembly (Supplementary Fig. 1a). The MrC26 genome had the highest overall number of variants when compared with the other genotypes, as well as the highest number of genotype-specific variants (Supplementary Fig. 1b). The MrCO84 and MrE7 isolates were the most closely related, with the lowest number of variant bases between them and the highest number of shared SVs when compared with the other isolates (Supplementary Fig. 1a and b).

**Fig. 2.**
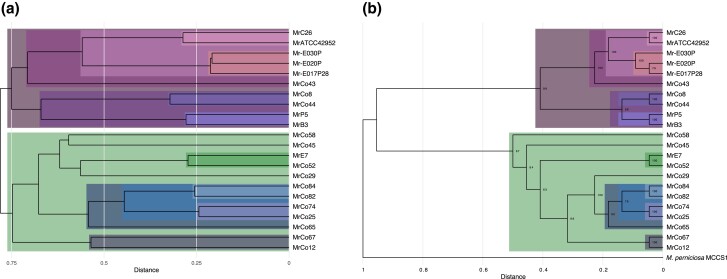
Phylogenetic trees relating the *M. roreri* isolates. Phylogenetic trees describing the relationships present between the *M. roreri* isolates (A and A) and *M. perniciosa* (B). Relationships identified with both methods have been highlighted with the same color. a) Tree built on the binary distance between isolates according to the presence of shared breakpoints of the SVs identified between genomes. b) Phylogenetic tree based on amino acid sequence conservation among the single-copy orthologous proteins.

Comparisons of breakpoint/rearrangement patterns between Illumina sequences for 22 isolates and individual PacBio assemblies show that similar SVs are present in independently derived isolates of *M. roreri*. A distance matrix, based on the presence or absence of SVs, was constructed for all isolates and used to generate dendrograms using each individual SMRT assembly as a reference (Supplementary Fig. 2). This was followed by a combined analysis using breakpoints across all SMRT assemblies (Fig. 2a). These dendrograms show a strong correspondence with the phylogenetic relationships between isolates determined through the multiple alignment of 1,445 single-copy genes (Fig. 2b) and with the synonymous groups identified through SNP analysis of the 22 isolates (Ali *et al.* 2015). In fact, the Illumina sequences of 6 isolates that were considered unique in the earlier SNP study (Ali *et al.* 2015) were also identified as unique based on scaffold breakpoints and single gene similarities.

### Impact of structural variation on the gene space

**Fig. 3.**
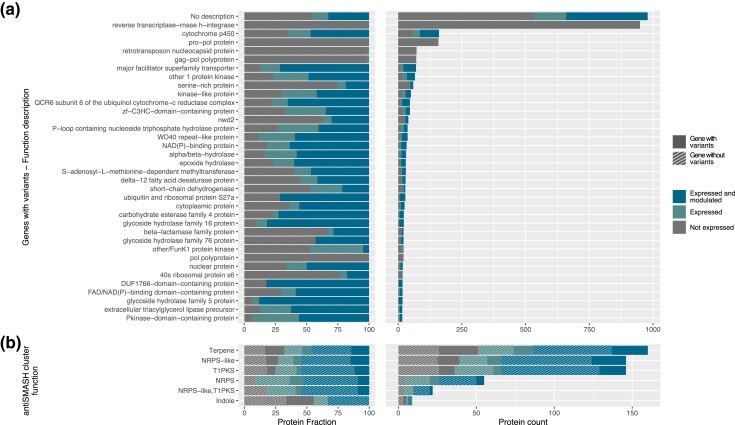
Function of genes affected by SNPs and SVs. a) Distribution of the gene functions associated with the genes affected by SVs in long-read assemblies when comparing MrC26 with the other 4 isolates. Most of the genes are associated with transposable elements, no homolog in RefSeq database, or no biological function, often related to fungal pathogenicity (Pusztahelyi *et al.* 2015; Macheleidt *et al.* 2016). Expression and modulation in the RNA-Seq samples have been also reported, showing that many of the genes related to the secondary metabolism are active, with DE meaning significantly differentially expressed in at least 1 condition, EX meaning expressed in at least 1 condition even if not significantly modulated, and NO meaning not expressed. b) Secondary metabolite clusters found in MrC26 with antiSMASH. Expression and modulation in the RNA-Seq samples have been also reported as long as the presence of SVs in the locus.

Clustering of the *M. roreri* proteins annotated in all 22 isolates identified 23,437 orthogroups. Of these clusters, 62.2% (14,576) were conserved across all isolates and consisted of single-copy genes, while 1,102 were found to have a variable number of copies (Supplementary Fig. 4). The remaining 7,759 groups were not shared between all isolates (Supplementary Fig. 3), with 408 groups being specific to a single isolate. The variability in gene content confirms the significant impact of structural variability on the gene space (Fig. 3a). When comparing the SVs to the gene annotation, the highest number of genes affected by variants were associated with transposable elements (e.g. reverse transcriptase-rnase h-integrase, gag−pol polyprotein, retrotransposon nucleocapsid protein, and pol polyprotein, Supplementary Fig. 5), further emphasizing the major role of TE mobility in the variation of the genomic structure of *M. roreri* isolates. Other major categories affected by structural variability were associated with secondary metabolism (e.g. cytochrome p450), cellular transport (e.g. MFS transporters), and cell wall modifications (Carbohydrate-Active Enzymes, CAZymes, Supplementary Fig. 6), which may play a role in pathogenicity. RNA-Seq analysis of MrC26 showed that most of these genes were also expressed and modulated across different sampling conditions (Fig. 3a and Supplementary Fig. 7). The results of our secondary metabolites cluster analysis provided a similar observation. We found that the long-read assembled genomes exhibited an exceptional level of consistency in terms of functional categories, cluster number, and cluster size (Supplementary Fig. 8). However, the impact of SVs was evident in several clusters, affecting all functional categories that we identified. In particular, we found that a significant number of expressed and modulated genes were affected by these SVs (Fig. 3b).

**Fig. 4.**
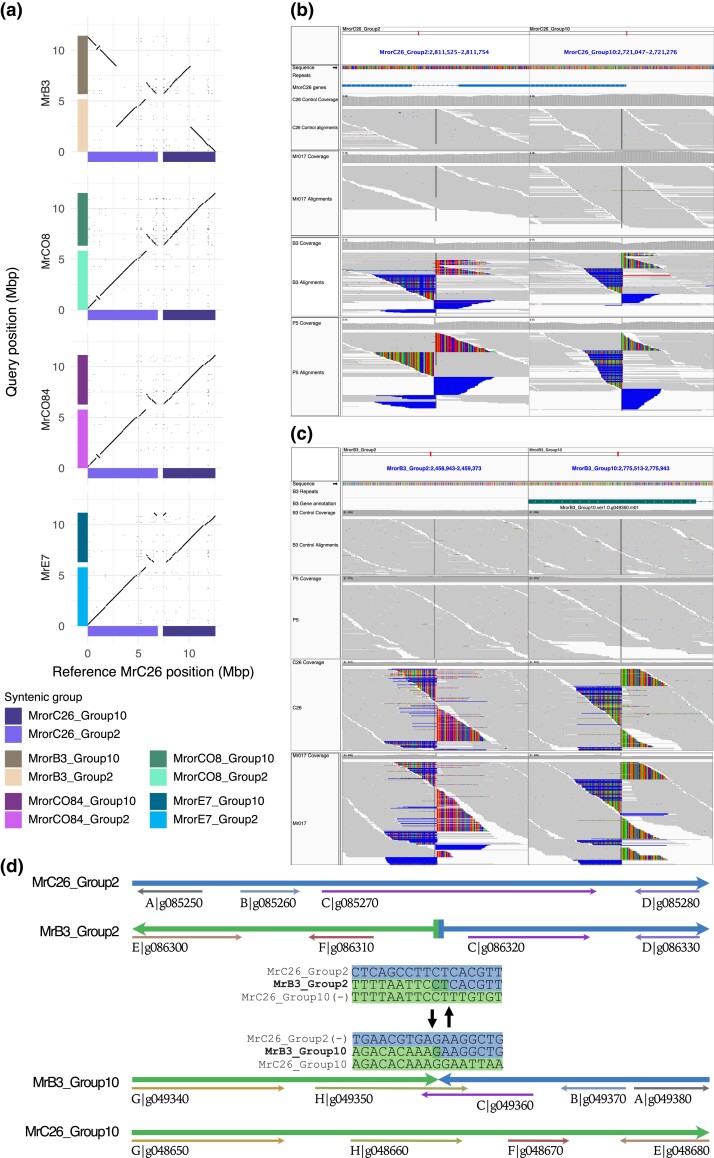
SVs identified between syntenic groups 2 and group 10 in the different *M. roreri* isolates. Syntenic group 2 and syntenic group 10 show the largest SV identified among the *M. roreri* isolates assembled with long reads. a) The dotplots evidence the presence of a translocation event specific of MrB3 and a duplication event specific of MrC26 among the 5 long-read assemblies. b) and c) Raw read alignments confirm the translocation event in MrB3 and evidence that is shared only with the closely related isolate MrP5 using both MrC26 (b) and MrB3 (c) genome assemblies as reference. d) Detailed view of the translocation breakpoint and the effect on the gene content of MrC26 and MrB3.

Syntenic groups 2 and 10 exhibit some of the most notable SVs across the different isolates. A large translocation is present in the MrB3 isolate between syntenic groups 2 and 10, as compared with the other 4 *M. roreri* isolates (Fig. 4a). This was verified through the detection of identical breakpoints/sequence in contigs from independently obtained short sequence assemblies and in the sequenced reads (Fig. 4b).

Specifically, MrP5 and MrB3 share the same breakpoint, while MrC26, MrAT42952, and all other isolates do not show evidence of this translocation event. The SV appears to have originated in the ancestors of MrB3 and causes a modification of the gene content of the locus by breaking in a gene-rich area (Fig. 4c). RNA-Seq conducted on MrC26 showed that the genes around the breakpoint are expressed (Supplementary Table 4). Additionally, the MrC26 genome shows a duplication between syntenic groups 2 and 10 (Fig. 4a). This duplication involves 2 32,849 bp tandem repeats at the terminal end, composed of retroelement sequences as well as at least 290 non–retroelement-related genes, at least 140 of which are expressed. This results in the duplication of 15 secreted proteins, 10 of which are candidate effectors. Two of these are unique to the *Marasmiaceae*, and 2 are unique to *M. roreri* and *M. perniciosa* (Mr/Mp) among the comparisons made. One Mr/Mp-unique candidate effector, an adhesin protein called Mad1-related protein, is preferentially expressed during the biotrophic phase.

### Candidate effector profiles

**Fig. 5.**
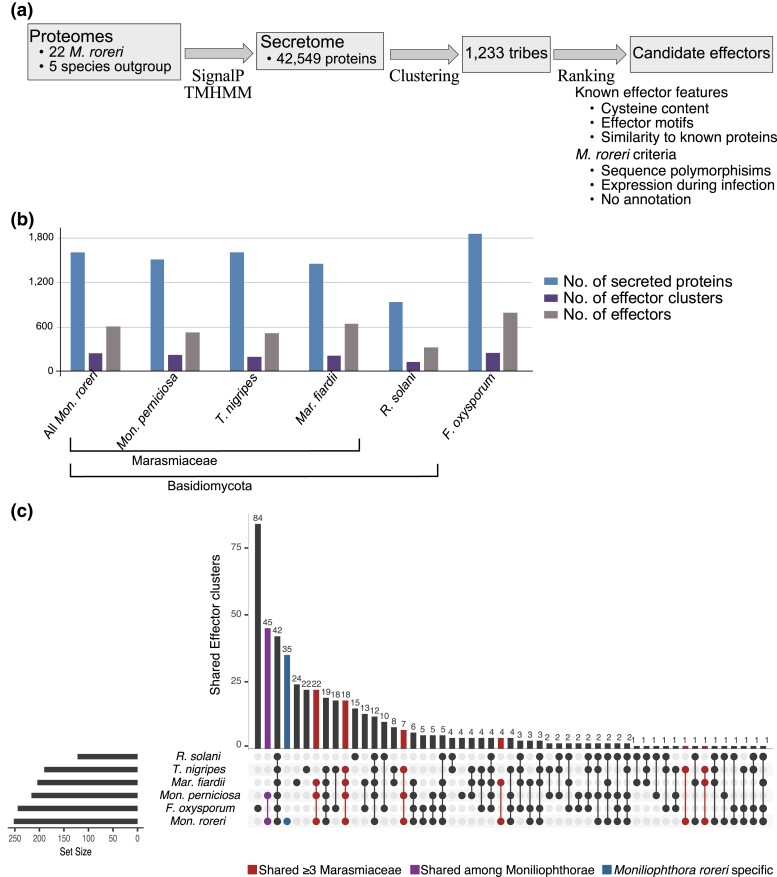
Secreted protein in *M. roreri*. a) Secretome identification pipeline used for *M. roreri*. b) Number of secreted proteins, effectors, and effector clusters identified in the 22 *M. roreri* isolates, in the *M. perniciosa* Mp-MCCS1 isolate, and in the species used as outgroup (*F. oxysporum forma specialis Lycopersici* 4286, *M. fiardii* PR-910, *R. solani* AG-1 IA, and *T. nigripes* CBS291.85), c) Effector clusters shared across the different species; the effectors specific of *M. roreri* are evidenced in blue, the effector clusters shared between the *Moniliophthorae* in violet, and the effector clusters shared between *M. roreri* and at least 1 other *Marasmiaceae* species with or without *M. perniciosa* and not *F. oxysporum* or *R. solani* in red.

A search for effectors identified an average of 1,601.1 ± 16.9 secreted proteins for each of 22 *M. roreri* genomes (Fig. 5a and b and Supplementary Tables 5 and 6). Among the secreted proteins, an average 739.5 ± 9.5 for each *M. roreri* isolate was classified as candidate effectors. Analysis of the effector sequences in the *M. roreri* isolates and 6 other species identified 263 effector tribes in *M. roreri*. Of these, 53 tribes were unique to the 4 *Marasmiaceae* (including *M. roreri* and 1 other *Marasmiaceae* species other than *M. perniciosa*), 45 tribes were unique to *M. roreri* and *M. perniciosa* (Mr/Mp), and 35 tribes were unique to *M. roreri* only (Fig. 5c). Genes with upregulated expression at 60 days compared with 30 DPI were enriched in secreted genes, effector genes, and *Marasmiaceae*-specific effector genes (Fisher's exact test, *P* ≤ 0.005; Table 2). None of the *Marasmiaceae*-specific effector genes were upregulated at 30 DPI compared with 60 DPI. Genes upregulated at 30 DPI compared with 60 DPI were only enriched in Mr/Mp-specific effector genes (Table 2). The small pool of Mr-specific effector genes did not show a preference for up- or downregulation at either phase of infection (Table 2).

**Table 2.** Expression modulation of secreted genes during biotrophic or necrotrophic phases.

Subset*^a^*	Phase*^b^*	In subset		Not in subset		Odds ratio estimate*^c^*	
		Upregulated	Not modulated	Upregulated	Not modulated		*P*-value*^d^*
Secreted proteins							
(1622 genes)	Biotrophic	62	1,339	824	17,770	1.00	1
	Necrotrophic	221	1,339	1,244	17,770	2.36	<2.2 × 10^−16^
Effector proteins							
(599 genes)	Biotrophic	25	493	861	18,616	1.10	0.664
	Necrotrophic	81	493	1,384	18,616	2.21	2.874 × 10^−9^
*M. roreri* secreted proteins							
(33 genes)	Biotrophic	2	30	884	19,079	1.44	0.652
	Necrotrophic	1	30	1,464	19,079	0.43	0.723
*M. roreri and M. perniciosa* secreted proteins							
(74 genes)	Biotrophic	10	55	876	19,054	3.95	5.428 × 10^−4^
	Necrotrophic	9	55	1,456	19,054	2.14	0.0455
*Marasmiaceae* secreted proteins							
(97 genes)	Biotrophic	0	77	886	19,032	0.00	0.051
	Necrotrophic	20	77	1,445	19,032	5.58	1.058 × 10^−8^

The modulation of 5 different subsets of secreted proteins is compared with the rest of the annotated genes in MrC26 isolate (21,460 annotated gene loci) during the transition between biotrophic phase (30 DPI) and the necrotrophic phase (60 DPI) in cocoa-infected pods. *^a^*Subsets include all secretome genes, predicted effectors, secreted genes unique to *M. roreri* (Mr), secreted genes in both *M. roreri* and *M. perniciosa* (Mr/Mp), or secreted genes in *Marasmiaceae* (Mar)—the latter 3 categories are mutually exclusive. *^b^*Phase indicates upregulated at 30 DPI vs 60 DPI (Biotrophic) or upregulated at 60 DPI vs 30 DPI (Necrotrophic). *^c^*Values >1 and <1 indicate the proportions of genes in the subset upregulated are greater or less than the proportions of genes not in the subset upregulated, respectively. *^d^P*-values < 0.05 are in bold.

Among the genes in the *Marasmiaceae*-specific effector pool, 20 were upregulated at 60 DPI (Table 2). These necrotrophy-specific genes encode components of the fungal cell surface or enzymes expected to modify surfaces. Of the 74 genes in the MrC26/Mp-specific gene pool, 10 were upregulated at 30 DPI compared with 60 DPI, while 9 genes were upregulated at 60 DPI (Table 2). The biotrophic phase–specific genes encode putative adhesion-related proteins, a Pr-1-like protein, an immunomodulatory protein, several unknown proteins, and others. The necrotrophic-specific genes encode putative mucin-2-like isoform X2-related proteins and a necrosis-inducing-like protein (npp1), among others. Only 2 of the 33 MrC26-specific candidate effector genes (MrorC26_Group4.ver1.0.g127640.m01 and MrorC26_Group9.ver1.0.g204140.m01, genes predicted to encode a fungal hydrophobin and an integral membrane protein, respectively) were upregulated at 30 days vs 60 days, and 1 of the 33 MrC26-specific candidate effector genes (MrorC26_Group10.ver1.0.g055790.m01, a gene predicted to encode a cytochrome P450) was upregulated at 60 days vs 30 days (Table 2).

### Identification of new mating type loci variants

**Fig. 6.**
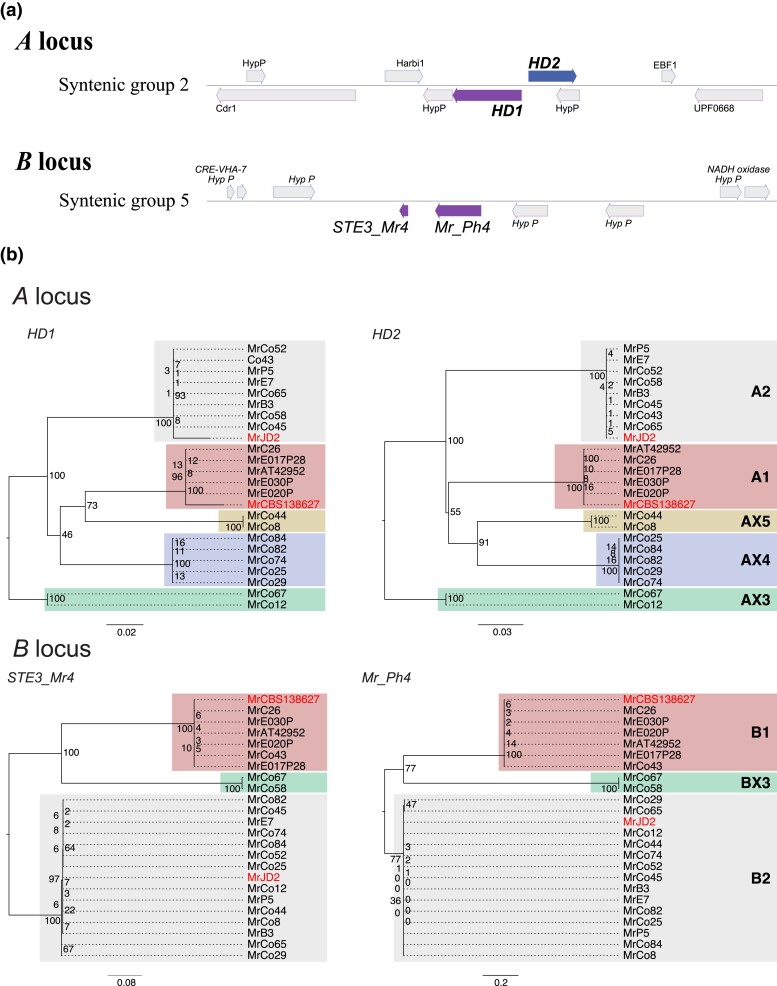
Phylogeny of mating type loci. a) Location and structure of mating type loci. *Moniliophthora roreri* mating type locus A genes (HD1 and HD2) are located in syntenic group 2, and locus B genes (STE3_Mr4 and Mr_Ph4) are located in syntenic group 5. b) Phylogenetic trees describing the relationships present between the mating type genes identified in the 22 *M. roreri* isolates. The HD1 and HD2 (A locus) and STE3_Mr4 and Mr_Ph4 (B locus) proteins previously described in Díaz-Valderrama and Aime (2016a) for A1B1 and A2B2 mating types are evidenced in red. A1, A2, and alleles for A1 locus were assigned using HD1 and HD2 gene sequence homology, and X3, X4, and X4 IDs were assigned to the newly identified alleles. B locus alleles B1 and B2 were assigned based on homology of STE3_Mr4 and Mr_Ph4 genes alleles, and the newly identified locus was denominated BX3.

**Fig. 7.**
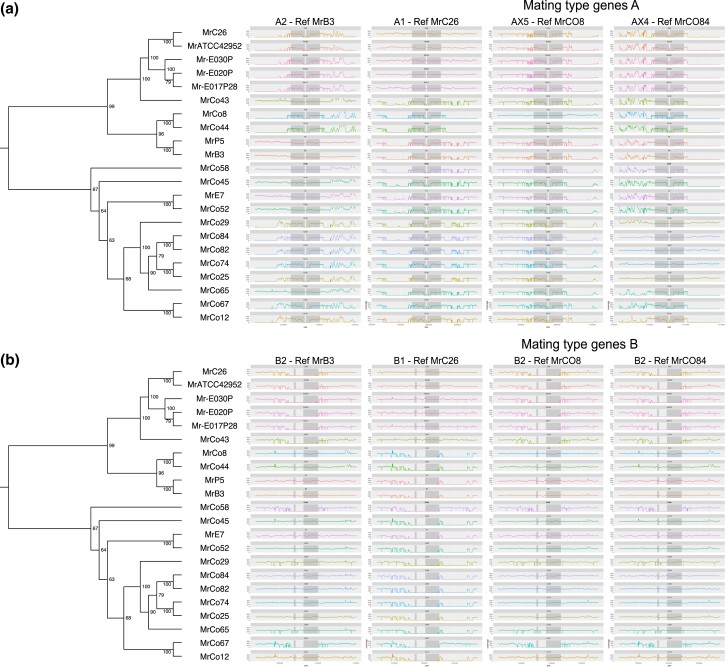
*Moniliophthora roreri* mating type loci variability across 22 isolates. Comparison of mating type loci structure across different isolates in relation to the phylogeny calculated on the conservation of single-copy ortholog proteins. By aligning the raw Illumina reads of each isolate on the different A locus structures (a) and B locus structures (b) identified for the long-read assemblies, it is possible to identify the closest allele structure. The location of both genes of each mating type locus is highlighted in gray in the plot. B locus shows a lower amount of variation among the isolates and the presence of 3 different alleles, and A locus counts 5 alleles that do closely follow the relationships described by the single-copy orthologs.

**Fig. 8.**
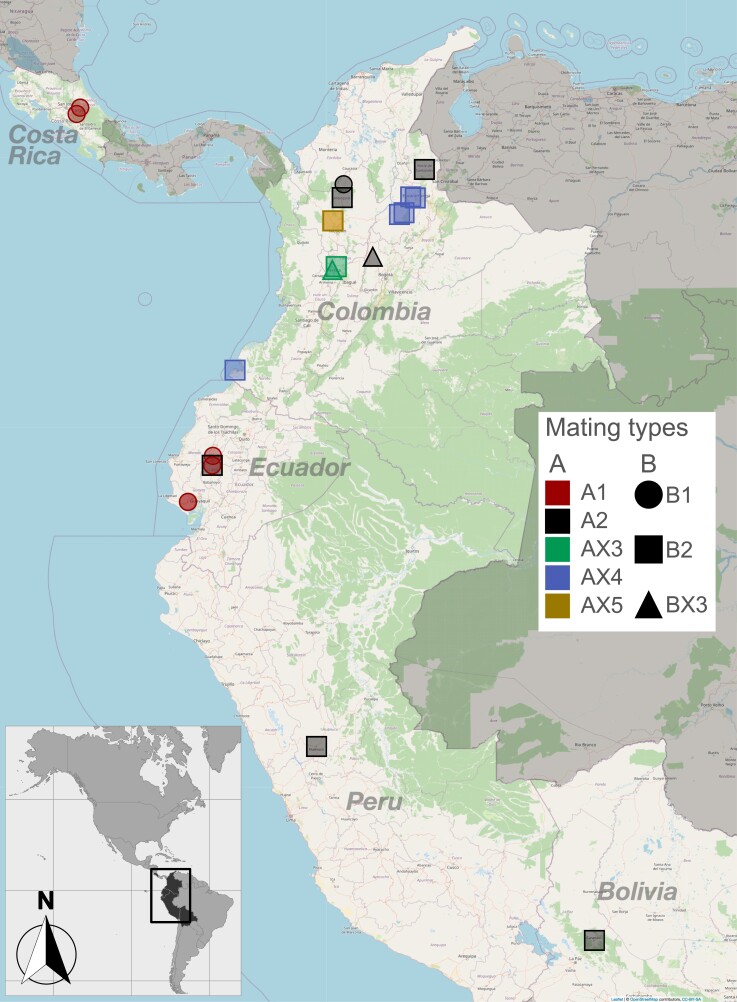
*Moniliophthora roreri mating* type loci allele geographical distribution. Geographical distribution of the different mating type alleles. Boxes sharing the same color carry the same mating type alleles. Color coding has been used to distinguish the mating type A locus alleles (A1, A2, and the newly identified AX3, AX4, and AX5); icon shape was used to distinguish the B locus alleles (B1, B2, and the newly identified BX3).

Despite being asexual and haploid, *M. roreri* carries the genes required for tetrapolar mating at the A and B loci, located on syntenic group 2 and group 5, respectively (Fig. 6a). The A locus consists of 2 consecutive genes, HD1 and HD2, arranged in opposite orientation with neighboring gene starts in the middle of the locus (Fig. 6a). Variation between alleles is primarily located along the 2 start sequences (Fig. 7a). The B locus also consists of 2 consecutive gene loci, Ph4 and STE3, both oriented on the negative strand of the syntenic group 5 (Fig. 6a), with sequence variation extending across both gene sequences (Fig. 7b). The regions bordering the 2 mating type loci showed close similarity among isolates carrying the same allele, but varied between isolates carrying different alleles, with the degree of dissimilarity depending on the alleles being compared (Fig. 7b). Two allelic configurations were previously reported for *M. roreri*, namely A1B1 and A2B2 (Díaz-Valderrama and Aime 2016a). In our population, 5 isolates found between Costa Rica and Ecuador could be assigned to the A1B1 allelic form (Fig. 8); 6 isolates spreading across Colombia, Ecuador, Peru, and Bolivia could be assigned to the A2B2 allelic form (Fig. 8). A single A2B1 isolate was identified in Colombia (Fig. 8). By assembling the genomes of all isolates in our population, we were able to identify 3 previously unreported alleles for the A locus (called AX3, AX4, and AX5) and a new allele for the B locus (called BX3). Notably, the BX3 locus was not identified by gene prediction tools during annotation procedure, the locus manually identified through a focused search of an open reading frame in the expected genomic region. The isolates carrying the newly identified alleles for the A locus (2 AX3 isolates, 5 AX4 isolates, and 2 AX5 isolates) and the 2 isolates carrying the BX3 allele were collected in Colombia. The similarity of the alleles of each gene loci is shown in Fig. 7b. After analyzing the sequence similarity among single-copy genes across their genomes (Fig. 7b), the 5 A1B1 isolates from the far ends of the pathogen's range (Costa Rica and Ecuador) showed some diversity but still clustered together. Isolates carrying the A2B2 allelic combination showed varied groupings, intersecting with other A locus allelic variants but excluding the B1 allele (Fig. 6b).

## Discussion

*Moniliophthora roreri* is thought to have evolved in the mountain valleys of Colombia and Ecuador, possibly on *Theobroma* species other than cacao (Evans 2007, 2016a). An alternative hypothesis suggests it originated in the upper Amazon and was introduced to Colombia and Ecuador (Díaz-Valderrama *et al.* 2022). *Moniliophthora roreri* continues to spread to new areas, including Jamaica (Johnson *et al.* 2017) and western Brazil (da Silva *et al.* 2022). The timing of *M. roreri*'s transition from sexual to asexual reproduction remains unclear. History, dating back as far as 1817 in Colombia, has always described *M. roreri* based on the asexual spores produced on pods (Phillips-Mora 2003; Evans 2016a). These spores, rhexolytic thallic conidia, are the primary means of infection and dispersal of *M. roreri* (Díaz-Valderrama and Aime 2016b). While some conidia contain only 1 haploid nucleus, most have 2 or more identical homokaryotic haploid nuclei formed from multinucleated mycelia, which are derived from mycelia that are typically mononucleated (Bailey, Ali *et al.* 2018). Multiple genome sequence assemblies consistently show the absence of heterozygosity in the *M. roreri* genome (Meinhardt *et al.* 2014; Díaz-Valderrama and Aime 2016a).

### Structural changes in the *M. roreri* genome in association with geographic dispersion

The ability of pathogens to adapt to changes in their hosts through genetic modification is critical to maintaining pathogen/host relationships (Karasov *et al.* 2014). Sexual reproduction generates new genotypes through recombination while maintaining species identity through maintenance of chromosome structure (Heng 2007). On the other hand, homothallism and asexual reproduction contribute to genome evolution and speciation through accumulation and stabilization of chromosome modifications and repetitive sequence (Whittle *et al.* 2011). The genome of *M. roreri* was estimated to be 52 Mbp with 13.5% of repetitive content (Meinhardt *et al.* 2014). Our multiple single-molecule real-time long-read sequences assemblies of the *M. roreri* genome revealed an average genome size of nearly 58 Mbp with 11 scaffolds (Table 1). The number of *M. roreri* chromosomes may be smaller than the number of scaffolds identified. Karyotyping estimated the chromosome number of *M. perniciosa* to be between 8 and 10 for cacao isolates (Rincones *et al.* 2006), but karyotyping has not been performed on *M. roreri*. The genome of *M. roreri* shows an accumulation of repeats across multiple isolates (>17.5%), and multiple isolate-specific scaffold rearrangements (Table 1 and Fig. 1). The repeat sequences are organized into relatively dense blocks with little associated functional gene structure. This is quite different than in some pathogens, for example, the *Phytophthoras*, where repeat sequence can be extensive and associated with increased numbers of virulence-associated genes (Poplin *et al*. 2017; Morales-Cruz *et al.* 2020).

The rearrangements, found and validated among 5 SMRT assemblies, include translocations, duplications, and inversions (Supplementary Fig. 1). The scaffold rearrangements were verified by comparison of breakpoints in multiple Illumina sequence assemblies of related isolates (Supplementary Fig. 2). The large reciprocal translocation between syntenic groups 2 and 10 found in isolates B3 as compared with other isolates is a unique finding (Figs. 2 and 4). Isolate MrB3 was previously placed into synonymous group 2 (Ali *et al.* 2015), the only synonymous SNP group found in Ecuador without a representative found in Colombia. Synonymous group 2 was composed of isolates from a single isolate from Manabi, Ecuador, and multiple isolates from Peru and Bolivia at the southern edge of *M. roreri* range. Breakpoints associated with the reciprocal translocation were shared with Peruvian isolate MrP5 (also synonymous group 2).

MrC26 is representative of the narrow genetic diversity found throughout Central America (Ali *et al.* 2015; Díaz-Valderrama *et al.* 2022) at the northern edge of *M. roreri* range and falls within synonymous group 1 which includes members from Colombia and Ecuador. The MrC26 genome assembly is unusual in that it carries a duplicated segment on the end of syntenic group 2 originating from syntenic group 10 (Fig. 4). It is through duplications and deletions that gene composition may be most directly altered. This is the case in MrC26, as the duplicated segment includes more than 270 potentially functional genes along with a large tandem repeat of retroelement-associated sequence.

### New mating type variants were identified in Colombia

Forced asexual reproduction can result in genome degradation in sexual fungi (Horgen *et al.* 1996; Saleh *et al.* 2012) and loss of sexual reproduction in nature. For example, Saleh *et al.* (2012) suggested the movement of *Magnaporthe oryzae* into new areas resulted in increased asexual reproduction due to mating type isolation and permanent loss of sexual reproduction potential outside its Asian center of origin. Due to the discontinuous distribution of *Theobroma* species in their natural habitats, both *M. roreri* and *M. perniciosa* were likely under pressure to eliminate or simplify their tetrapolar mating system (Kües and Navarro-González 2010; Díaz-Valderrama and Aime 2016a). In *M. roreri*, the structures of the unlinked mating type locus A (syntenic group 2) and B (syntenic group 5) have been described in detail (Díaz-Valderrama and Aime 2016a). Díaz-Valderrama and Aime (2016a) found only 2 mating type combinations (A1B1 and A2B2) in their initial characterization of the *M. roreri* mating gene loci, but sampling inside Colombia was limited to 5 isolates from a single region, Antioquia. Forty-one isolates from outside Colombia were included in the study with 10 isolates from a total of 6 locations in Ecuador (Díaz-Valderrama and Aime 2016a). The isolates sequenced in the current study were selected from observed *M. roreri* SNP diversity (Ali *et al.* 2015) originally including more than 60 isolates collected throughout Colombia as well as 11 isolates from Ecuador and additional isolates from Peru, Bolivia, and Venezuela. We identified 3 new alleles (5 alleles total) for the A locus and 1 potentially new allele (3 alleles total) for the B locus (Figs. 7 and 8) occurring in 8 AB locus allelic combinations. All the A and B locus allele combinations differing from the A1B1 and A2B2 combinations were found within Colombia (Fig. 6). This supports Colombia as a center of diversity and possible center of origin for *M. roreri* and establishes the possibility of a more complex historical mating system in *M. roreri* than originally proposed (Díaz-Valderrama and Aime 2016a).

Although we have 5 A locus alleles and 3 B locus alleles, most of the non-A1B1 mating type allele combinations we identified show potential for combining with only the A1B1 variant in a traditional tetrapolar mating system, A2B1 and AX3BX3 isolates being exceptions. The BX3 allele of the B locus was not called by gene annotation software and may represent the nonfunctional evolution of the locus. This is interesting as Díaz-Valderrama and Aime (2016a) highlighted observations that in the Agaricomycetes bipolarity has always emerged because of “loss of mating type specificity and polymorphism in the *B* mating locus.” The identification of a single A2B1 isolate, something not observed by Díaz-Valderrama and Aime (2016a), at least alludes to possible sexual recombination in the past or at least some form of recombination. The 2 mating loci are on different scaffolds and would be expected to sort independently sexual reproduction processes. The observation that MrC26 (A1B1) and MrE7 (A2B2) share an inversion located on syntenic group 2 (Fig. 4) suggests they share lineages despite the different mating types providing additional evidence of recombination in the past having taken place after the inversion.

Currently, all *M. roreri* isolates collected outside Colombia are considered A1B1 or A2B2 [here and Díaz-Valderrama and Aime (2016a)]. In many places, we can clearly identify the time frame of the movement into new areas (Evans 2016a): for example, Costa Rica (all A1B1) in the late 1970s and in Peru in the late 1980s (both A1B1 and A2B2, Díaz-Valderrama and Aime 2016a) and in Bolivia (A2B2) by at least 2015 (Phillips-Mora *et al.* 2015). Note the 3 A1B1 isolates from Ecuador (Supplementary Table 1) all have SNP profiles matching synonymous group 4 (Ali *et al.* 2015), a group with isolates from both Ecuador and Colombia, while the 2 A1B1 isolates from Costa Rica fall within synonymous group 1, a group having isolates from Colombia, Costa Rica, and Ecuador (Ali *et al.* 2015). It is also worth noting isolate MrAT42952 was collected from Costa Rica in 1978 and shares genome structure (Fig. 2), locus sequence (Fig. 7), and SNP profile (Supplementary Table 1) with isolate MrC26 collected in Costa Rica 21 years later. Mating type variants other than A1B1 and A2B2 have been found close to the Colombia border with Ecuador in the Tumaco, Narino region (Fig. 6c), and it is likely additional mating type variants will be found outside and, possibly, within Colombia in the future (Espinoza-Lozano *et al.* 2022).

### Unique effector candidates show increased regulation in response to the biotrophic/necrotrophic shift

The manuscript describing the first *M. roreri* genome focused on the secretome (Meinhardt *et al.* 2014) identifying 1,535 candidate genes in a 52.3 Mb genome. Some genes differentially expressed between the biotrophic/necrotrophic phases were discussed as potential effectors: Pr1-like, cerato-platanins, and “biotrophy-associated” secreted proteins. Subsequent work by Barbosa *et al.* (2018) identified 243 candidate effectors in a 45.2 Mb *M. roreri* genome composed of 2,994 contigs, but lacked expression data. Here, multiple genome assemblies of *M. roreri* have identified a large number of candidate effectors, with estimated in the range from 1,535 to 2,994. Our highly contiguous genome assembly identified 1,622 candidate secreted proteins and 599 candidate effectors, using a combination of different prediction tools and focusing on small, secreted proteins with high cysteine content. The genome analysis identified numerous potential effector proteins that are unique to *M. roreri*, *M. perniciosa*, or *Marasmiaceae* species. Some of these proteins show preferential expression during the biotrophic or necrotrophic phases. Many of the proteins that show differential expression between the biotrophic and necrotrophic phases are associated with the cell surface or surface modification. For example, Pr-1-like proteins and necrosis-inducing protein-like Npp1s may be important in plant–pathogen interactions and have been found to be under positive selection in the *Moniliophthoras* (Vasconcelos *et al.* 2021) or to have been acquired through gene transfer (Tiburcio *et al.* 2010), respectively. It appears many genes encoding secreted proteins/candidate effectors are downregulated during the biotrophic phase, potentially limiting pathogen recognition, but are upregulated during the necrotrophic phase to contribute to tissue necrosis and pathogen growth. On the other hand, a unique set of genes encoding candidate effectors in *M. roreri* and *M. perniciosa* are upregulated during the biotrophic phase, potentially contributing to the avoidance or overcoming of plant defense responses, allowing for the establishment of infections and early tissue colonization. It is notable the large segment duplication found in the genome of MrC26 carries 10 candidate effectors including 2 effectors unique to Mr/Mp (Fig. 5). These unique genes, an undescribed gene and a Mad1-like adhesin protein, were upregulated in the biotrophic phase. Adhesins function in the adhesion of spores to plants and insects (Epstein and Nicholson 2006) and may respond to exudates from their host in the context of the prolonged *M. roreri* biotrophic phase. A GH18 protein which was previously identified as an active effector in *M. roreri* (Fiorin *et al.* 2018) was not identified as a candidate effector by our structure-based screening approach. This finding indicates that the identification of fungal effectors remains challenging due to their structural diversity.

In conclusion, the current research presents 4 focused and unique findings. We assembled 5 near-complete *M. roreri* genome assemblies allowing new discussion of the pathogen's genome evolution. A maximum of 11 scaffolds are indicated, although the chromosome number may be smaller. *Moniliophthora roreri* isolates are accumulating chromosomal rearrangements, changes in repeat sequence composition, and changes in independent line of descent clonal lineages. Scaffold rearrangements identified include large and small segmental translocations and duplications between and within scaffolds, along with inversions. We also identify multiple additional mating type alleles expanding what was considered 2 mating type allele pattern combinations (A1B1 and A2B2). A total of 5 A locus alleles and 3 B locus alleles were identified. The new mating types identified were found in isolates from Colombia, a proposed center of diversity for the pathogen. The use of multiple genome assemblies enabled us to broaden the pool of candidate effectors beyond what was previously estimated. Moreover, they provided evidence that genes encoding unique effector candidates for the *Moniliophthora* species have been preferentially expressed during the biotrophic phase of the disease. At the same time, genes encoding many secreted proteins and candidate effectors are repressed during the biotrophic phase, possibly to avoid pathogen recognition. Taken together, the clonal propagation of *M. roreri*, combined with its haploid genome, has allowed lineages with unique genome structures and gene compositions to dominate in areas as it expands its range displaying a significant founder effect.

## Supplementary Material

jkad125_Supplementary_Data

## Data Availability

Supplemental files are available at figshare: https://doi.org/10.25387/g3.22713796. Supplementary Fig. 1 contains the variant distribution across the 5 *M. roreri* SMRT-read assemblies, Supplementary Fig. 2 contains the phylogeny between *M. roreri* isolates based on shared SVs, Supplementary Fig. 3 contains the breakdown of non–single-copy genes shared between the different isolates, Supplementary Fig. 4 contains the shared orthogroups between the 22 *M. roreri* isolates, Supplementary Fig. 5 contains the counts of genes assigned to different CAZymes families and subfamilies, Supplementary Fig. 6 contains the counts of genes affected by variants categorized by gene function, Supplementary Fig. 7 contains the function distribution of the genes affected by SNPs and structural variants in MrC26, Supplementary Fig. 8 contains the comparison of secondary metabolites clusters identified in the different isolates, Supplementary Table 1 contains the geographic origin of the *M. roreri* isolates used in this study, Supplementary Table 2 contains the complete statistics for the assembly results, Supplementary Table 3 reports the genome assembly completeness assessment, Supplementary Table 4 reports the results of the RNA-Seq and differential expression analysis, Supplementary Table 5 reports the count and expression analysis results for the secretome genes, and Supplementary Table 6 contains the secretome gene cluster analysis results. Raw sequencing data were deposited at NCBI under BioProject number PRJNA925193, and RNA-Seq expression analysis results were deposited to GEOarchive under the accession numbers GSE226814 and GSE226813 for *M. roreri* and *M. perniciosa* respectively. Genome assemblies (**FASTA**), variant identified between accessions (**VCF**), gene annotation (**GFF3**), and repeat annotation (**GFF3**) files are deposited at Zenodo under the accession https://zenodo.org/record/7872498. .
