# Supplementary material for: Clonal reproduction of Moniliophthora roreri and the emergence of unique lineages with distinct genomes during range expansion
Source: G3 (Bethesda). 2023 Jun 20;13(9):jkad125. doi: 10.1093/g3journal/jkad125 (PMC10468315; doi:10.1093/g3journal/jkad125)
Supplement: jkad125_Supplementary_Data [file jkad125_supplementary_data.zip › Supplemental figure 2.pdf]

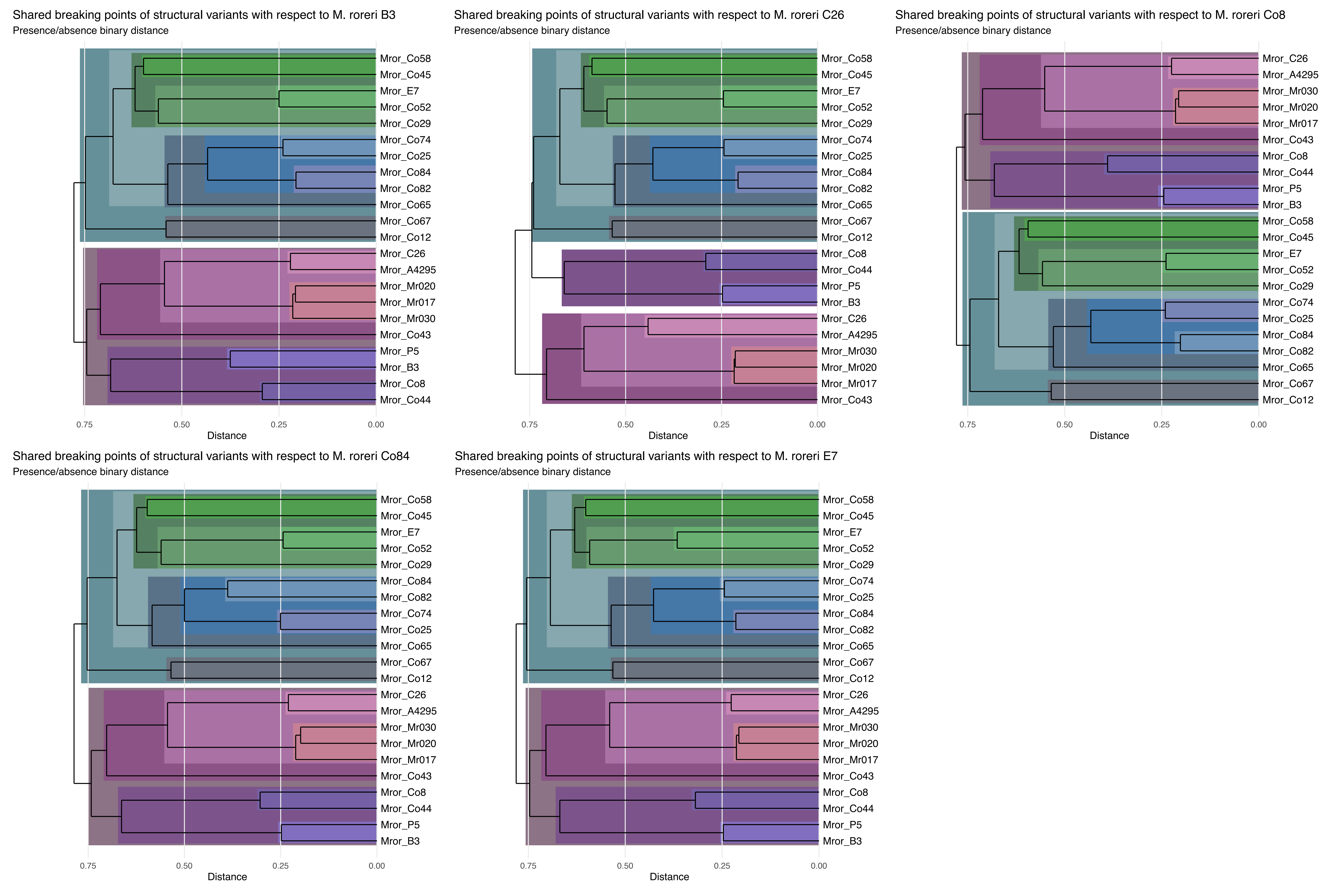
Shared breaking points of structural variants with respect to M. roreri B3
Presence/absence binary distance
Mror_Co58
Mror_Co45
Mror_E7
Mror_Co52
Mror_Co29
Mror_Co74
Mror_Co25
Mror_Co84
Mror_Co82
Mror_Co65
Mror_Co67
Mror_Co12
Mror_C26
Mror_A4295
Mror_Mr020
Mror_Mr017
Mror_Mr030
Mror_Co43
Mror_P5
Mror_B3
Mror_Co8
Mror_Co44
0.75
0.50
0.25
0.00
Distance
Shared breaking points of structural variants with respect to M. roreri C26
Presence/absence binary distance
Mror_Co58
Mror_Co45
Mror_E7
Mror_Co52
Mror_Co29
Mror_Co74
Mror_Co25
Mror_Co84
Mror_Co82
Mror_Co65
Mror_Co67
Mror_Co12
Mror_Co8
Mror_Co44
Mror_P5
Mror_B3
Mror_C26
Mror_A4295
Mror_Mr030
Mror_Mr020
Mror_Mr017
Mror_Co43
0.75
0.50
0.25
0.00
Distance
Shared breaking points of structural variants with respect to M. roreri Co8
Presence/absence binary distance
Mror_C26
Mror_A4295
Mror_Mr030
Mror_Mr020
Mror_Mr017
Mror_Co43
Mror_Co8
Mror_Co44
Mror_P5
Mror_B3
Mror_Co58
Mror_Co45
Mror_E7
Mror_Co52
Mror_Co29
Mror_Co74
Mror_Co25
Mror_Co84
Mror_Co82
Mror_Co65
Mror_Co67
Mror_Co12
0.75
0.50
0.25
0.00
Distance
Shared breaking points of structural variants with respect to M. roreri Co84
Presence/absence binary distance
Mror_Co58
Mror_Co45
Mror_E7
Mror_Co52
Mror_Co29
Mror_Co84
Mror_Co82
Mror_Co74
Mror_Co25
Mror_Co65
Mror_Co67
Mror_Co12
Mror_C26
Mror_A4295
Mror_Mr030
Mror_Mr020
Mror_Mr017
Mror_Co43
Mror_Co8
Mror_Co44
Mror_P5
Mror_B3
0.75
0.50
0.25
0.00
Distance
Shared breaking points of structural variants with respect to M. roreri E7
Presence/absence binary distance
Mror_Co58
Mror_Co45
Mror_E7
Mror_Co52
Mror_Co29
Mror_Co74
Mror_Co25
Mror_Co84
Mror_Co82
Mror_Co65
Mror_Co67
Mror_Co12
Mror_C26
Mror_A4295
Mror_Mr030
Mror_Mr020
Mror_Mr017
Mror_Co43
Mror_Co8
Mror_Co44
Mror_P5
Mror_B3
0.75
0.50
0.25
0.00
Distance
