# Supplementary material for: Clonal reproduction of Moniliophthora roreri and the emergence of unique lineages with distinct genomes during range expansion
Source: G3 (Bethesda). 2023 Jun 20;13(9):jkad125. doi: 10.1093/g3journal/jkad125 (PMC10468315; doi:10.1093/g3journal/jkad125)
Supplement: jkad125_Supplementary_Data [file jkad125_supplementary_data.zip › Supplemental figure 3.pdf]

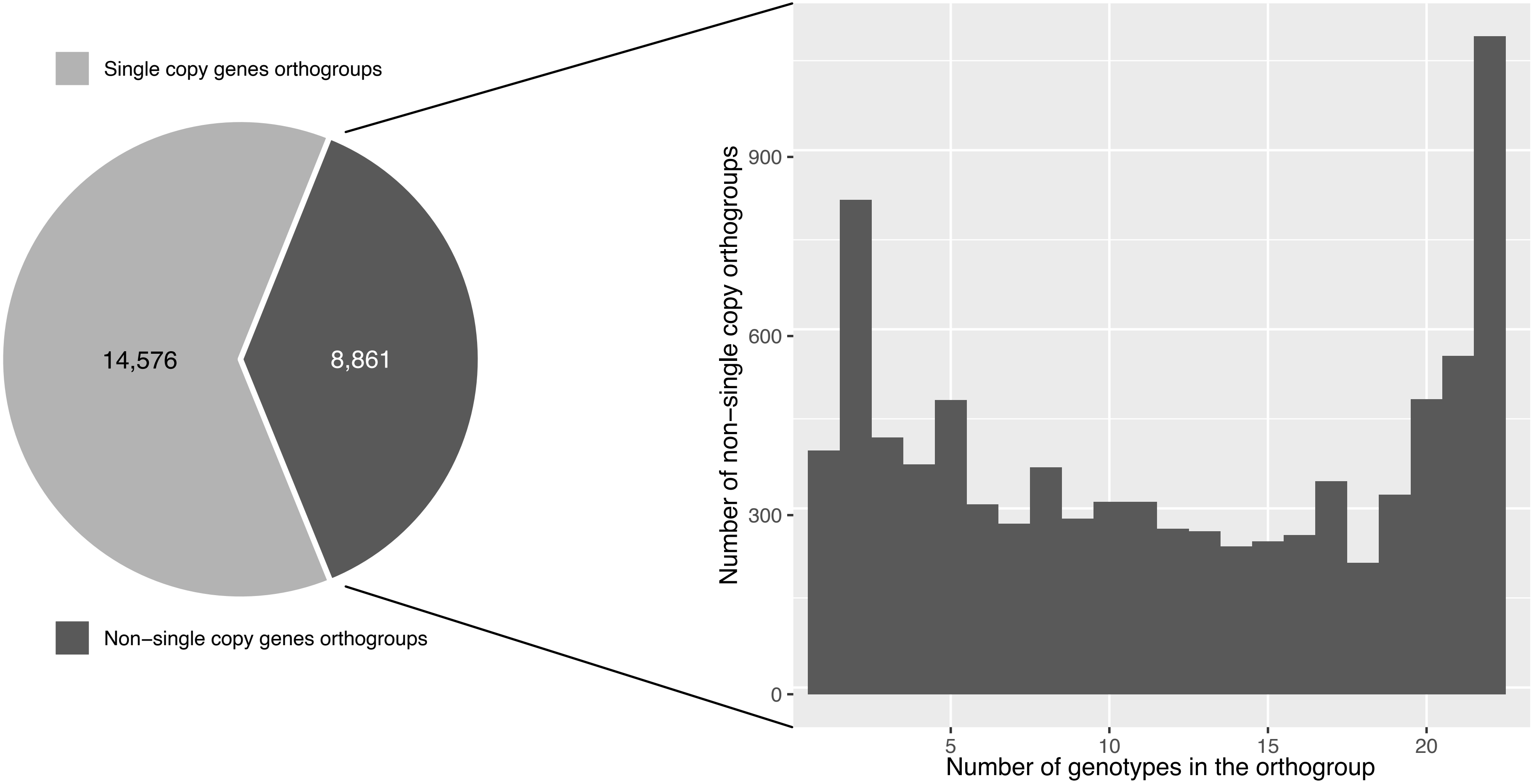

Single copy genes orthogroups
14,576
8,861
Non−single copy genes orthogroups
Number of non−single copy orthogroups
0
300
600
900
5
10
15
20
Number of genotypes in the orthogroup
