# Supplementary material for: Clonal reproduction of Moniliophthora roreri and the emergence of unique lineages with distinct genomes during range expansion
Source: G3 (Bethesda). 2023 Jun 20;13(9):jkad125. doi: 10.1093/g3journal/jkad125 (PMC10468315; doi:10.1093/g3journal/jkad125)
Supplement: jkad125_Supplementary_Data [file jkad125_supplementary_data.zip › Supplemental figure 5.pdf]

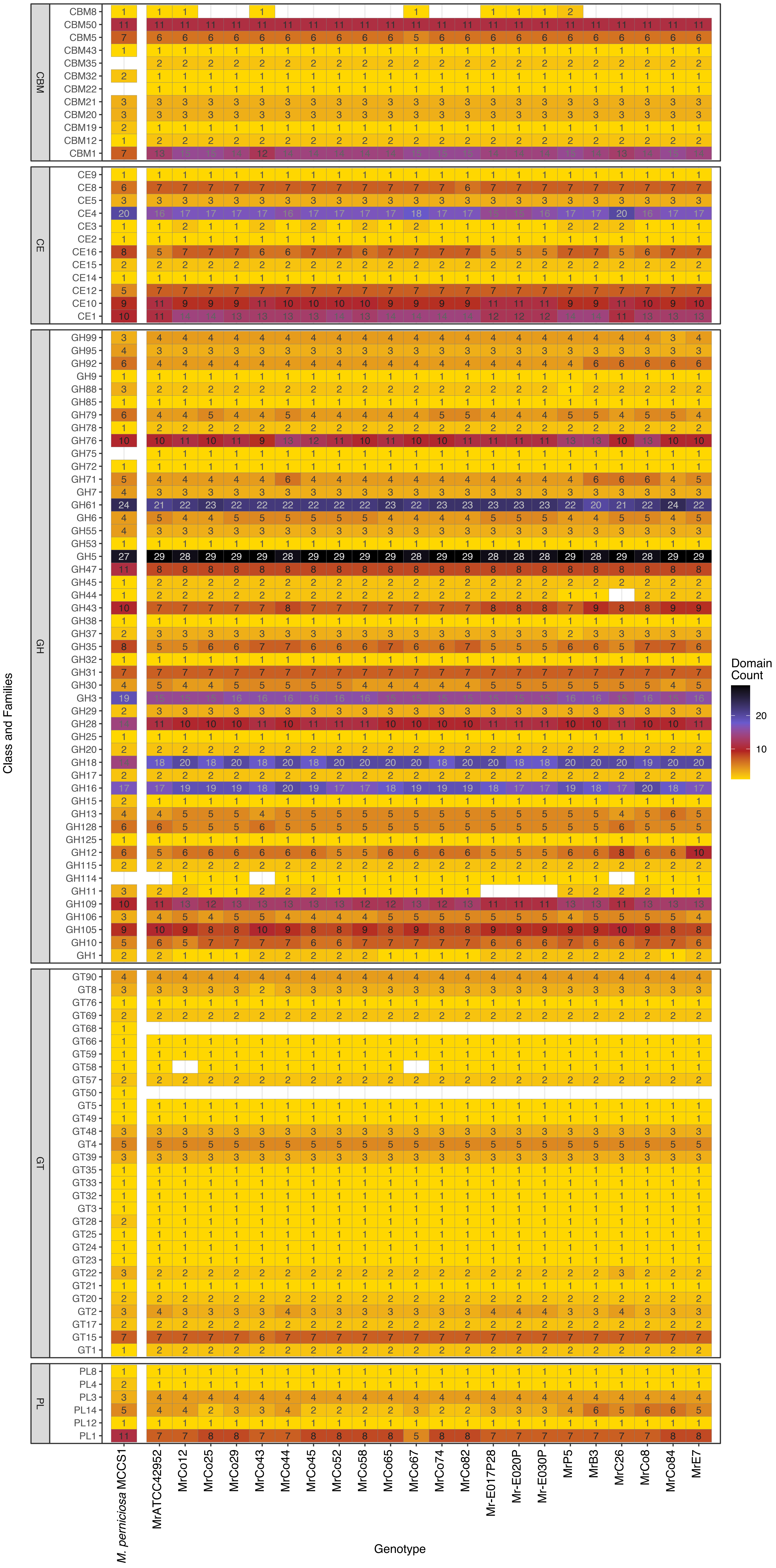
Class and Families
CBM
CBM8
CBM50
CBM5
CBM43
CBM35
CBM32
CBM22
CBM21
CBM20
CBM19
CBM12
CBM1
CE
CE9
CE8
CE5
CE4
CE3
CE2
CE16
CE15
CE14
CE12
CE10
CE1
GH
GH99
GH95
GH92
GH9
GH88
GH85
GH79
GH78
GH76
GH75
GH72
GH71
GH7
GH61
GH6
GH55
GH53
GH5
GH47
GH45
GH44
GH43
GH38
GH37
GH35
GH32
GH31
GH30
GH3
GH29
GH28
GH25
GH20
GH18
GH17
GH16
GH15
GH13
GH128
GH125
GH12
GH115
GH114
GH11
GH109
GH106
GH105
GH10
GH1
GT
GT90
GT8
GT76
GT69
GT68
GT66
GT59
GT58
GT57
GT50
GT5
GT49
GT48
GT4
GT39
GT35
GT33
GT32
GT3
GT28
GT25
GT24
GT23
GT22
GT21
GT20
GT2
GT17
GT15
GT1
PL
PL8
PL4
PL3
PL14
PL12
PL1
*M. perniciosa* MCCS1
MrATCC42952
MrCo12
MrCo25
MrCo29
MrCo43
MrCo44
MrCo45
MrCo52
MrCo58
MrCo65
MrCo67
MrCo74
MrCo82
Mr-E017P28
Mr-E020P
Mr-E030P
MrP5
MrB3
MrC26
MrCo8
MrCo84
MrE7
Genotype
Domain Count
20
10
