# Supplementary figures and images for: Clonal reproduction of Moniliophthora roreri and the emergence of unique lineages with distinct genomes during range expansion
Source: G3 (Bethesda). 2023 Jun 20;13(9):jkad125. doi: 10.1093/g3journal/jkad125 (PMC10468315; doi:10.1093/g3journal/jkad125)

A

Query

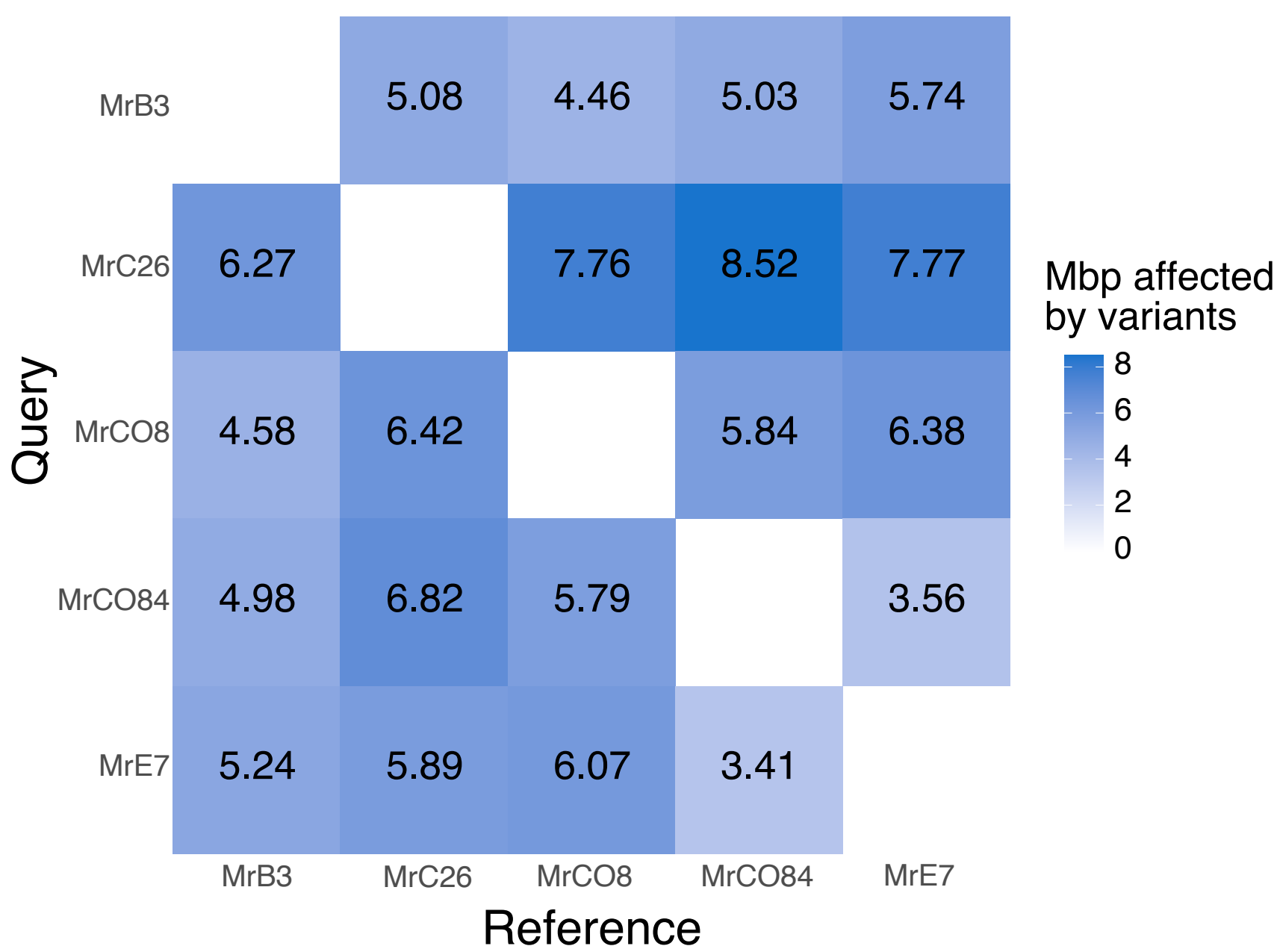

B

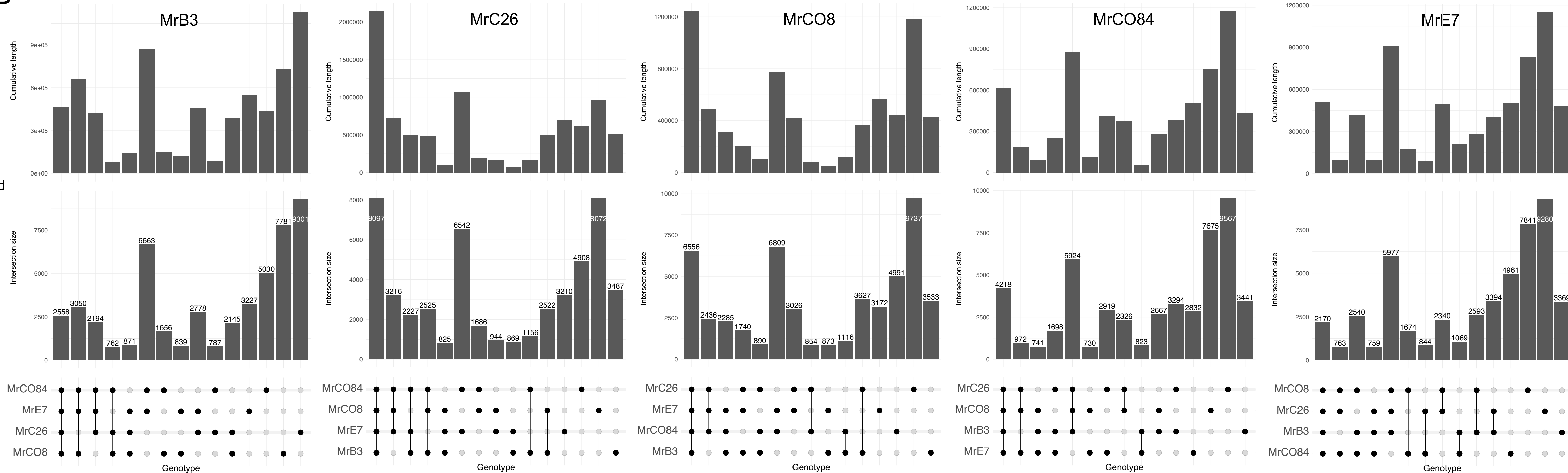

C

Query

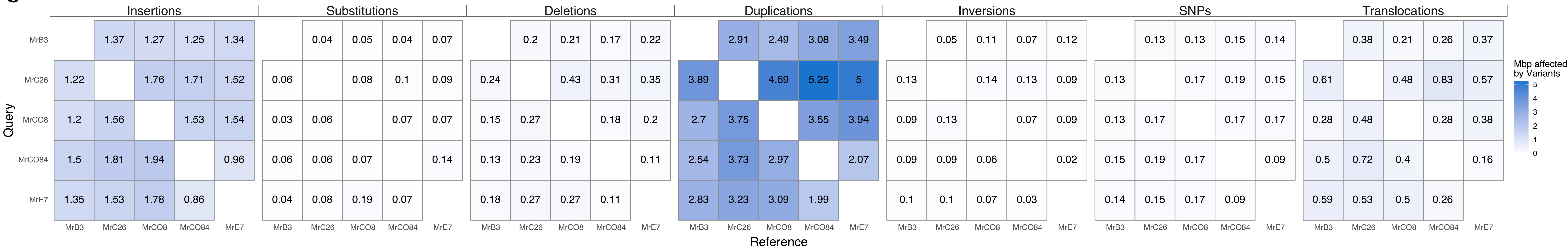

Supplement: jkad125_Supplementary_Data [file jkad125_supplementary_data.zip › Supplemental figure 1.pdf]

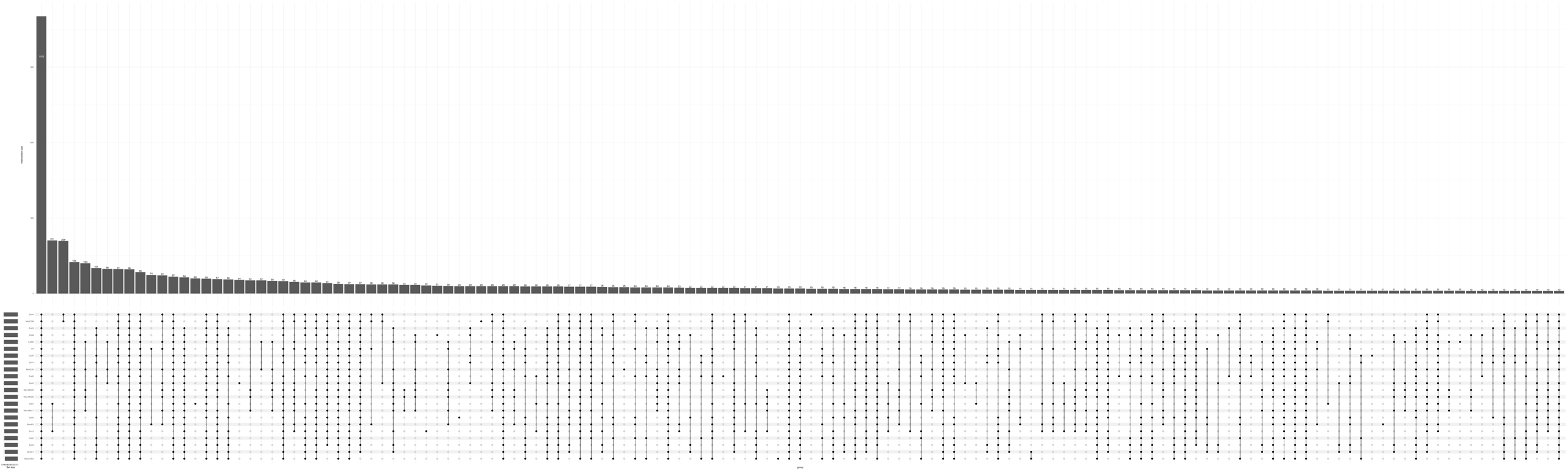

Supplement: jkad125_Supplementary_Data [file jkad125_supplementary_data.zip › Supplemental figure 4.pdf]

Genes affected by SVs

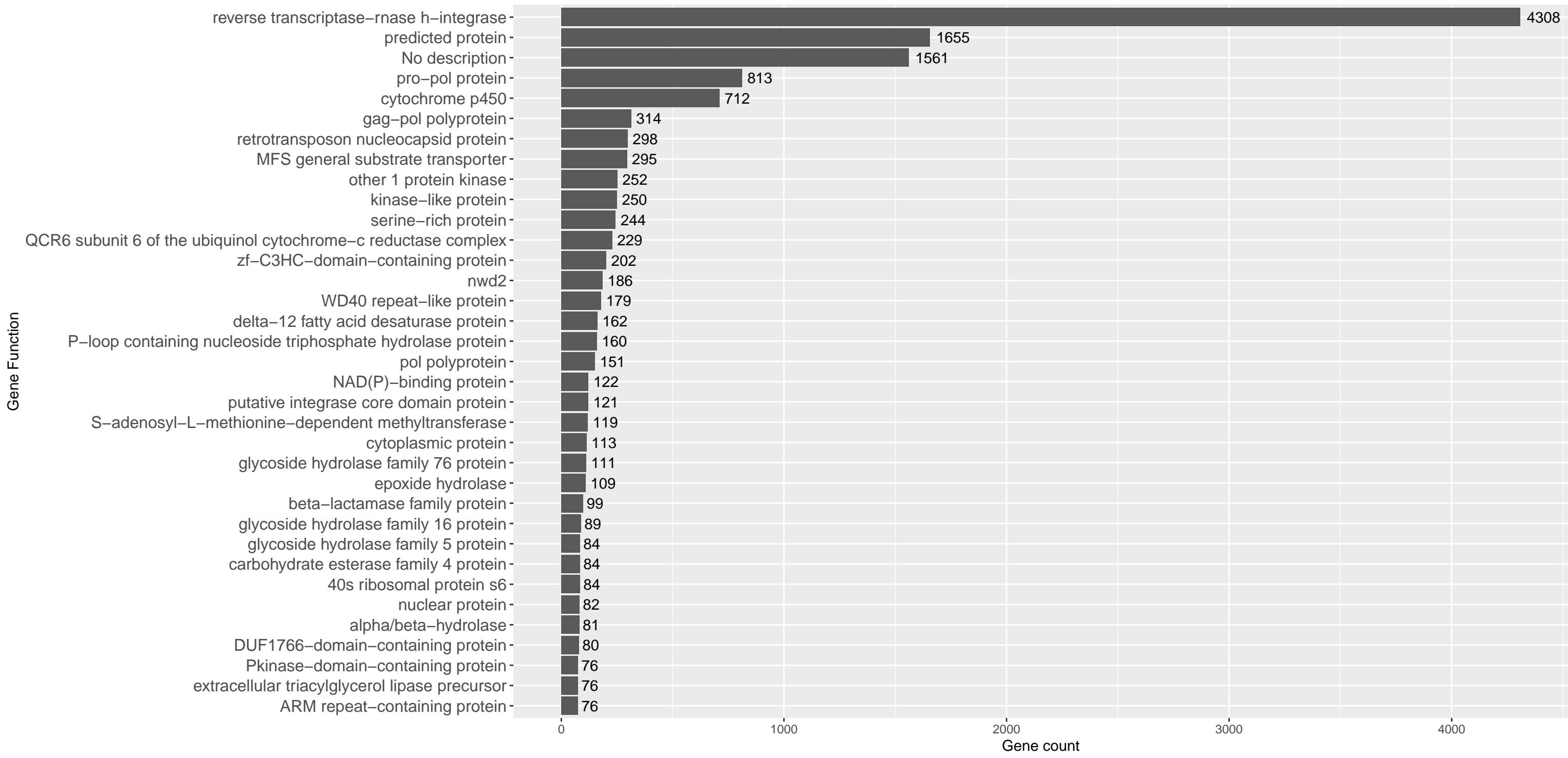

Supplement: jkad125_Supplementary_Data [file jkad125_supplementary_data.zip › Supplemental figure 6.pdf]

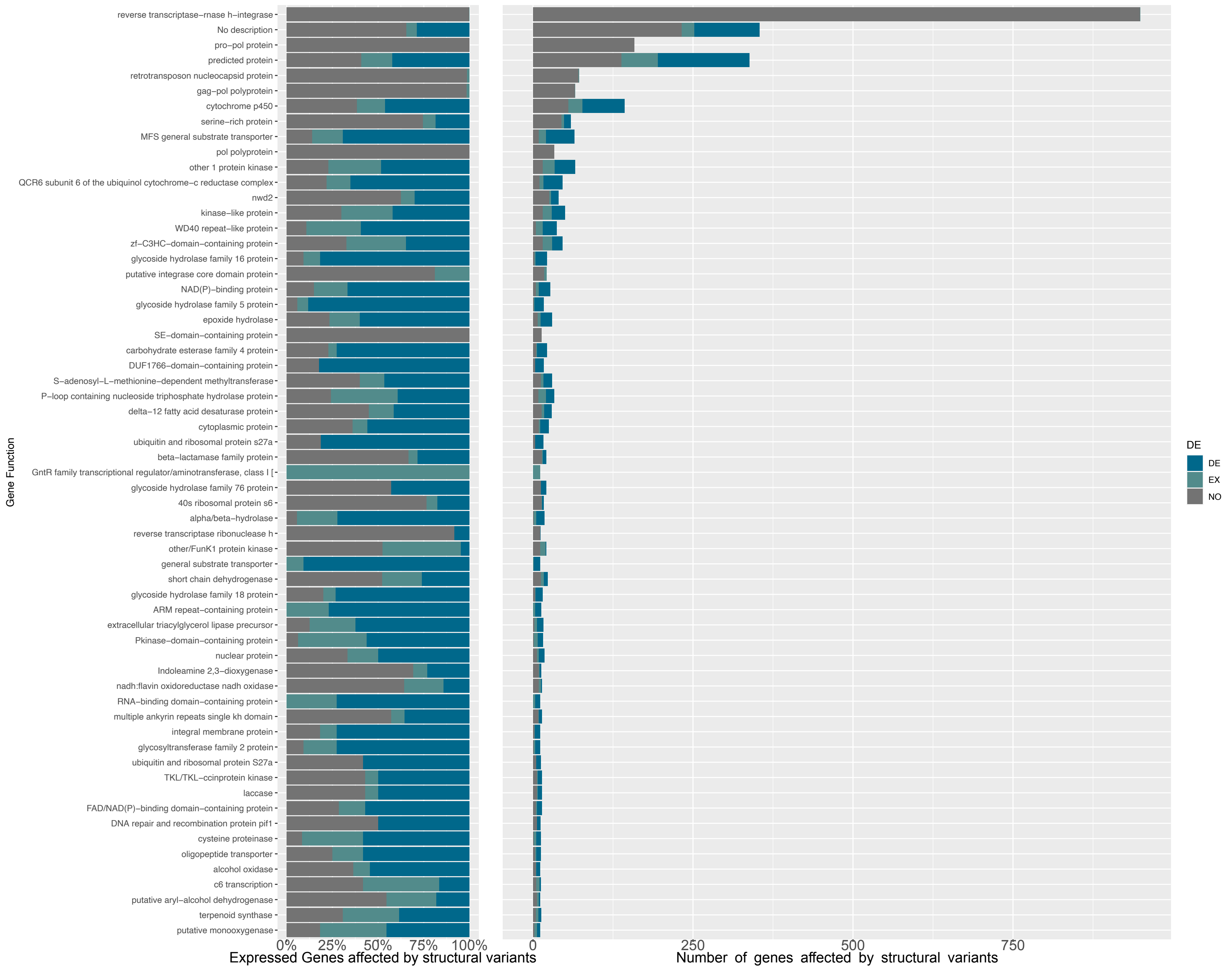

Supplement: jkad125_Supplementary_Data [file jkad125_supplementary_data.zip › Supplemental figure 7.pdf]
