# Supplementary material for: Clonal reproduction of Moniliophthora roreri and the emergence of unique lineages with distinct genomes during range expansion
Source: G3 (Bethesda). 2023 Jun 20;13(9):jkad125. doi: 10.1093/g3journal/jkad125 (PMC10468315; doi:10.1093/g3journal/jkad125)
Supplement: jkad125_Supplementary_Data [file Supplemental figure 8.pdf › Supplemental figure 8.pdf]

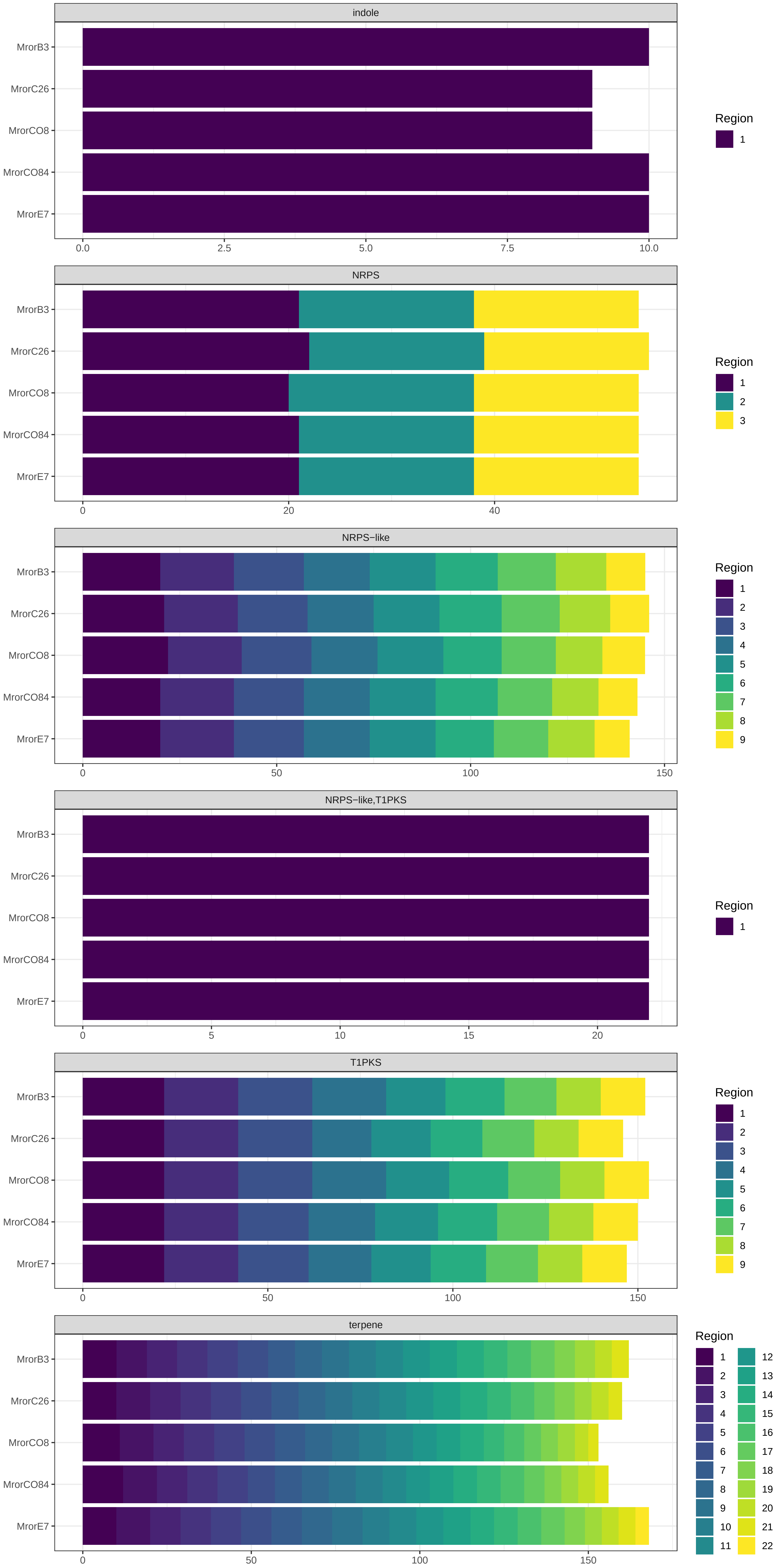
indole
NRPS
NRPS−like
NRPS−like,T1PKS
T1PKS
terpene
MrorB3
MrorC26
MrorCO8
MrorCO84
MrorE7
Region
